# Assessing hand motor function in chronic immune-mediated neuropathies: a proof-of-concept study using a data glove

**DOI:** 10.1186/s12984-024-01518-3

**Published:** 2024-12-20

**Authors:** Elisa Gilliam, Pascal Achenbach, Gernot J. Suemmermann, Manuel N. Wessely, Peter Rossmanith, Maike F. Dohrn, Jörg B. Schulz, Anne Waschbisch, Robert Brunkhorst

**Affiliations:** 1 Department of Neurology, RWTH Aachen University Hospital, Pauwelsstrasse 30, 52074 Aachen, Germany; 2 Institute of Neuropathology, RWTH Aachen University Hospital, Aachen, Germany; 3 Cynteract® GmbH, Aachen, Germany; 4 Theoretical Computer Science, Department of Computer Science, RTWH Aachen University, Aachen, Germany

**Keywords:** Data glove, Wearables, Chronic immune-mediated neuropathies, CIDP, MMN, Outcome measures, Nerve conduction studies, High-resolution ultrasound, Intravenous immunoglobulin, Rehabilitation

## Abstract

**Background:**

Chronic immune-mediated neuropathies are clinically heterogeneous and require regular, objective, and multidimensional monitoring to individualize treatment. However, established outcome measures are insufficient regarding measurement quality criteria (e.g., reliability, objectivity) or functional relevance. Wearables such as data gloves might be helpful, allowing repeated quantification of complex everyday life-relevant motor function of the hand.

**Methods:**

25 patients with chronic inflammatory demyelinating polyneuropathy or multifocal motor neuropathy were followed-up at five time points during maintenance therapy with intravenous immunoglobulin. 14 of them showed clinically relevant hand motor impairment. We examined the patients’ hand function using a data glove which quantifies the active range of motion (ROM) of the hand based on three different movement patterns*.* In addition, clinical outcome parameters (grip strength measurement, MRC Sum Score, INCAT disability score), nerve conduction studies (NCS), and high-resolution ultrasound (HRUS) were performed, and patient-reported outcome measures (PROMs) like the Rasch-built Overall Disability Scale (R-ODS) were assessed. We calculated correlation coefficients, performed Receiver Operating Characteristic analysis, as well as correlation analyses for the glove data and clinical outcome parameters. Longitudinal analyses were based on a Linear Mixed Model, and we assessed construct validity of the data glove by analyzing correlations between the glove measurements and well-established clinical parameters.

**Results:**

We found good to excellent test–retest reliability for the ROM in all glove movement patterns (Intraclass correlation coefficients = 0.83–0.94), underlining the ability to capture clinical stability. Moreover, the glove demonstrated adequate, sensitivity and specificity in detecting hand motor impairment (area under the curve (AUC): 0.714–0.780), and it performed better than NCS and HRUS (AUC: 0.552/0.701). The AUC values for the metrically scaled parameters include: Vigorimeter (AUC: 0.929) and R-ODS (AUC: 0.698). Additionally, the data glove proved to be a valid tool, as we demonstrated moderate to strong, significant correlations between the glove and established clinical parameters (especially Vigorimeter), as well as PROMs (especially R-ODS).

**Conclusions:**

This data glove allowed for a non-invasive assessment of the hand motor function and yielded investigator-independent results that reliably reflected individual functional deficits with relevance to everyday life. Future studies should explore the ability to predict clinically meaningful responses to immunomodulatory treatment and to support and monitor rehabilitation progress, with potential applications in other neurological diseases as well.

*Trial registration* at the German Clinical Trials Register, Deutsches Register Klinischer Studien (DRKS: 00027345), retrospectively registered on 23rd March 2022: https://drks.de/search/de/trial/DRKS00027345

**Supplementary Information:**

The online version contains supplementary material available at 10.1186/s12984-024-01518-3.

## Background

Patients diagnosed with chronic immune-mediated neuropathies, including chronic inflammatory demyelinating polyneuropathy (CIDP) or multifocal motor neuropathy (MMN), exhibit a variety of symptoms [1–3]. In addition to steroids, intravenous immunoglobulin (IVIg) therapy represents the primary treatment approach for these individuals [4, 5]. Due to high costs [6, 7], differing disease progression and responses to treatment [8], and individual pharmacokinetics [9, 10], dosage and treatment intervals need to be customized according to each patient's requirements [11, 12].

Given the variability in clinical presentation, optimal treatment decisions necessitate regular [8] and comprehensive evaluation, encompassing various outcome measures to address impairment, disability, symptoms, and Quality of Life (QoL) domains [1, 13, 14]. Frequently applied clinical outcome measures [1, 15] include grip strength (i.e., measured by the Martin Vigorimeter) [16], the Inflammatory Neuropathy Cause and Treatment (INCAT) disability score [17] and the Medical Research Council (MRC) Sum Score [18]. The Rasch-built Overall Disability Scale (R-ODS) [19, 20], the Beck Depression Inventory (BDI) [21], and the Fatigue Severity Scale (FSS) [22] are used as patient-reported outcome measures (PROMs). Quantitative and performance-based assessments include nerve conduction studies (NCS) [13] and high-resolution ultrasound (HRUS) [23, 24].

Unfortunately, not all the established instruments fully meet the required quality criteria, such as reliability and responsiveness [1, 15, 25]. Others are time-consuming or stressful for the patients (e.g. NCS[26]) and some either lack relevance to everyday life (e.g., MRC Sum Score) [27] or relevance for the individual patients (e.g., INCAT disability score) [1].

Shared decision-making is of uttermost importance in treating chronic diseases [28] such as CIDP, which is why PROMs are important tools to monitor the patient’s perception of the disease course [1, 13]. However, subjective impressions sometimes diverge from objectively recorded parameters, particularly in the context of treatment withdrawal [29, 30]. This often complicates treatment guidance and could be addressed by the implementation of wearables like data gloves, which might provide a sense of reassurance. Such tools hold the promise of enabling the quantitative evaluation of complex motor functions of the hand [31–33] by employing an objective and dynamic methodology [31, 33, 34]. Similar tools have already been applied to neurological diseases such as hereditary neuropathies [35], multiple sclerosis (MS) [36], and carpal tunnel syndrome (CTS) [37]. So far, however, the focus has primarily been on rehabilitation [38, 39]. The capability of data gloves for individual assessment and monitoring of patients with chronic immune-mediated neuropathies holds promise for the recognition of clinically relevant hand motor impairment. Furthermore, the identification of subtle changes in hand motor function could help to optimize treatment in patients with chronic immune-mediated neuropathies.

Here, we evaluated a new data glove as a diagnostic tool and disease activity monitoring outcome measure for patients with chronic immune-mediated neuropathies and relevant hand motor impairment.

## Methods and materials

### Study design, participants, and setting

For this prospective observational, non-interventional trial, 25 participants were recruited between March 11 and of May 11, 2022, from the neuromuscular outpatient clinic at the Department of Neurology, RWTH Aachen University Hospital, a designated center for chronic immune-mediated neuropathies. Data collection was completed on April 12, 2023. Prior to inclusion, patients gave written informed consent to participate in the study. The study was approved by the Ethics Committee of the University Hospital Aachen and was conducted in accordance with the Declaration of Helsinki.

Patients were eligible, if they had been diagnosed with CIDP or MMN according to the latest guidelines of the European Federation of Neurological Societies/Peripheral Nerve Society (EFNS/PNS) [4, 5]. They needed to be at least 18 years old and demonstrate clinical stability before enrollment, as determined by an experienced board-certified neurologist. Exclusion criteria included any confounding conditions that affect motor function of the hand (e.g., rheumatoid arthritis), other serious illnesses that would prevent participation in the study (e.g., dementia), and pregnancy.

Some patients with chronic immune-mediated neuropathies show motor impairment of the distal upper extremities, while others do not [2, 5]. At the initial timepoint, an experienced neurologist identified individuals with clinically relevant hand motor impairment to differentiate subgroups for subsequent analyses. This allocation was determined through a detailed neurological examination, with particular emphasis on motor functionality (including strength of thumb abduction, finger spread and hand extension) and aligned with clinical outcome parameters. The assessment of motor impairment focused on the dominant hand, or in cases of focal CIDP, the only affected hand, emphasizing the critical role of hand function in daily activities.

In this *proof-of-concept* study, we collected data during ongoing standard-of-care IVIg maintenance therapy. Patients were followed-up at five consecutive time points (T_0_ – T_4_). The interval between the different time points was determined by the individual treatment regimens.

To introduce the technique and to avoid confounding factors such as learning, the time point T_0_ was declared as a *learning time point* to familiarize with the assessments and especially with the glove. Consequently, we only included the data from T_1_-T_4_ in all subsequent analyses.

### Glove design and sensors

**Fig. 1:**
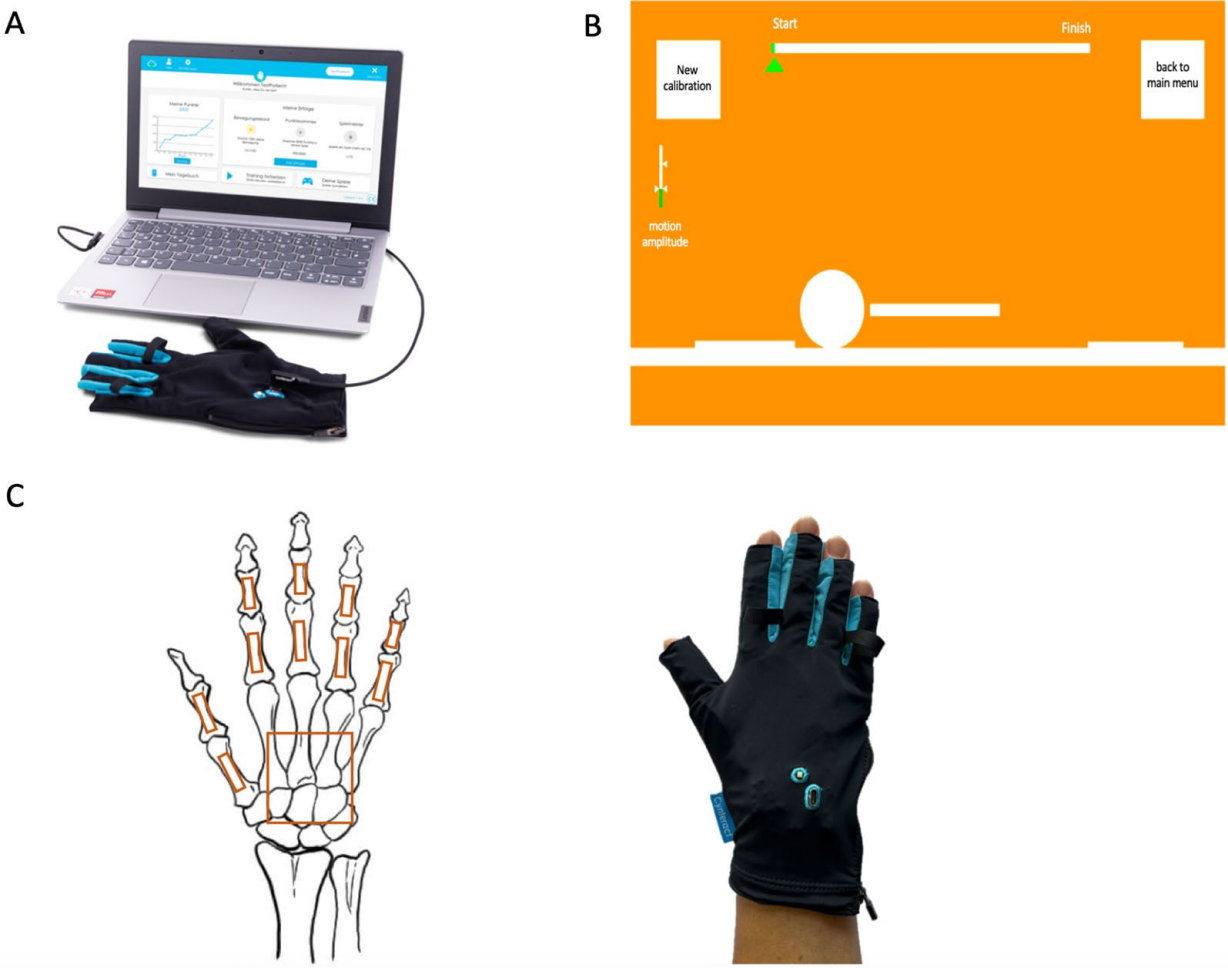
Overview on the technical and functional background of the glove. The data glove was connected to a computer/laptop running the corresponding software (**A**). Patients had to complete a course and its alternating obstacles by adjusting the size of a sphere, that was controlled by finger movements. To the left of the screen, a green bar indicated the current motion amplitude based on calibration. At the top, a progress bar specified the position within the course. The button in the upper right corner allowed for cancellation of the game and for returning to the main menu (**B**). Sensors for data acquisition were located over the dorsal sides of the proximal, resp., the middle phalanges of the long fingers. For the thumb, they were fixed at the first metacarpal bone and the proximal phalanx. The larger main board sensor was placed on the dorsum of the hand in the region of the metacarpus and the carpus and served as the central reference point for the proximal phalanges of the long fingers, as well as for the first metacarpal bone. Corresponding to the anatomical bone structures, on the right, the glove is shown as being worn (**C**)

Patients underwent repeated assessments with the data glove, manufactured by Cynteract® GmbH, Aachen, Germany at all consecutive time points. In combination with its corresponding software (Fig. 1), the glove is certified as a Class I Conformité Européenne (CE) medical device and measures the range of motion (ROM) of the hand.

The data glove consists of spandex material and weighs 60 g. It provides a USB cable connection to connect the glove to a laptop. For this study, there was one pair of unisize gloves for the left and the right hand.

To measure the angles of the finger joints, the data glove contains a total of eleven Bosch BNO055 nine-axis sensors that are sewn into the glove layers according to the hand anatomy (Fig. 1) and the sensors are connected by flexible silicone cables. A built-in accelerometer measures acceleration and a built-in gyroscope measures angular velocity. The sensor also embeds a magnetometer to measure the orientation of the sensor relative to the Earth´s magnetic field. It was switched off for this study due to interference from the clinical environment. Furthermore, each sensor contains an integrated processor that automatically fuses the data of the sensor components.

In the first step, the global rotation of each sensor is given as three-dimensional quaternions and is recorded approximately 25 times per second. From the quaternion information of two adjacent sensors, the angle of the finger joint situated in between is calculated in degrees (°). For this purpose, the rotation of the more distal sensor is relatively calculated to the rotation of the more proximal sensor.

Afterwards, the three-dimensional relative rotation is reduced to a specific rotation axis to determine a two-dimensional angle (in °). This axis varies depending on the particular type of angle (e.g., bending-/spread angle, or angle during opposition movement) of interest.

### Movement patterns of the data glove

**Fig. 2:**
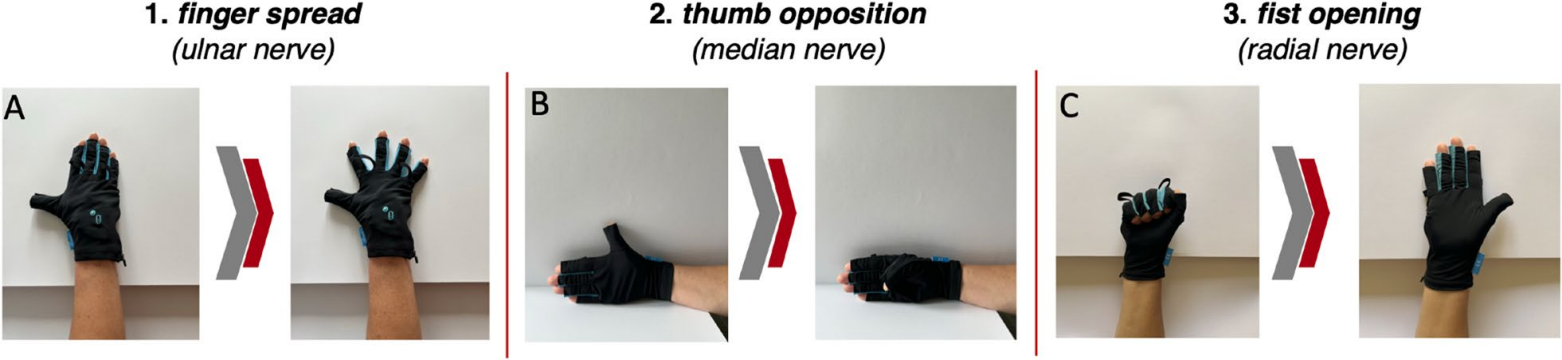
Overview on the three data glove movement patterns. The sequence of the three different movement patterns: *finger spread* (**A**), *thumb opposition* (**B**), and *fist opening* (**C**) is depicted. Since each of the three hand nerves controls only a specific movement direction, only that particular direction was included in the active range of motion (ROM) data analysis. The arrow between the right and left images indicates the movement direction, which is essentially mediated by one of the three hand nerves

We evaluated the active ROM of the hand using the data glove in three different glove movement patterns, referred to as *finger spread*, *thumb opposition* and *fist opening* (Fig. 2), out of which finger spread is mainly mediated by the ulnar nerve, thumb opposition by the median nerve, and the fist opening by the radial nerve. Thumb opposition is particularly important for daily tasks, such as grasping objects, and is considered one of the key aspects of hand motor function [37]. Additionally, finger spreading and fist opening also play essential roles in activities like gripping and manipulating various items. Videos of the movement patterns for better understanding can also be found by following the zenodo link (see Availability of data and materials).

The specific movements (Fig. 2), performed while wearing the data glove, control an interactive computer game. The computer game includes a standardized course of 50 alternating obstacles, which must be traversed by passing above or below the obstacles.

(Fig. 1). For this, the user had to adjust the size of a sphere by performing specifically defined hand movements in an alternating sequence.

Before the computer game starts, there is a *Reset* to determine the *zero-degree position*, that served as a reference for the subsequent angle determination (Additional file 1). To adapt the interactive game control to the individual ROM, the patients also need to *calibrate* the movement patterns before starting the course. Depending on the extent of hand motor impairment, a whole procedure takes approximately eight to 15 min per hand.

Moreover, we included a classical computer game called the *rocket game*, that was based on the movement pattern fist opening (Additional file 2) at the end of each study time point.

### Angle measurement and ROM analysis

**Fig. 3:**
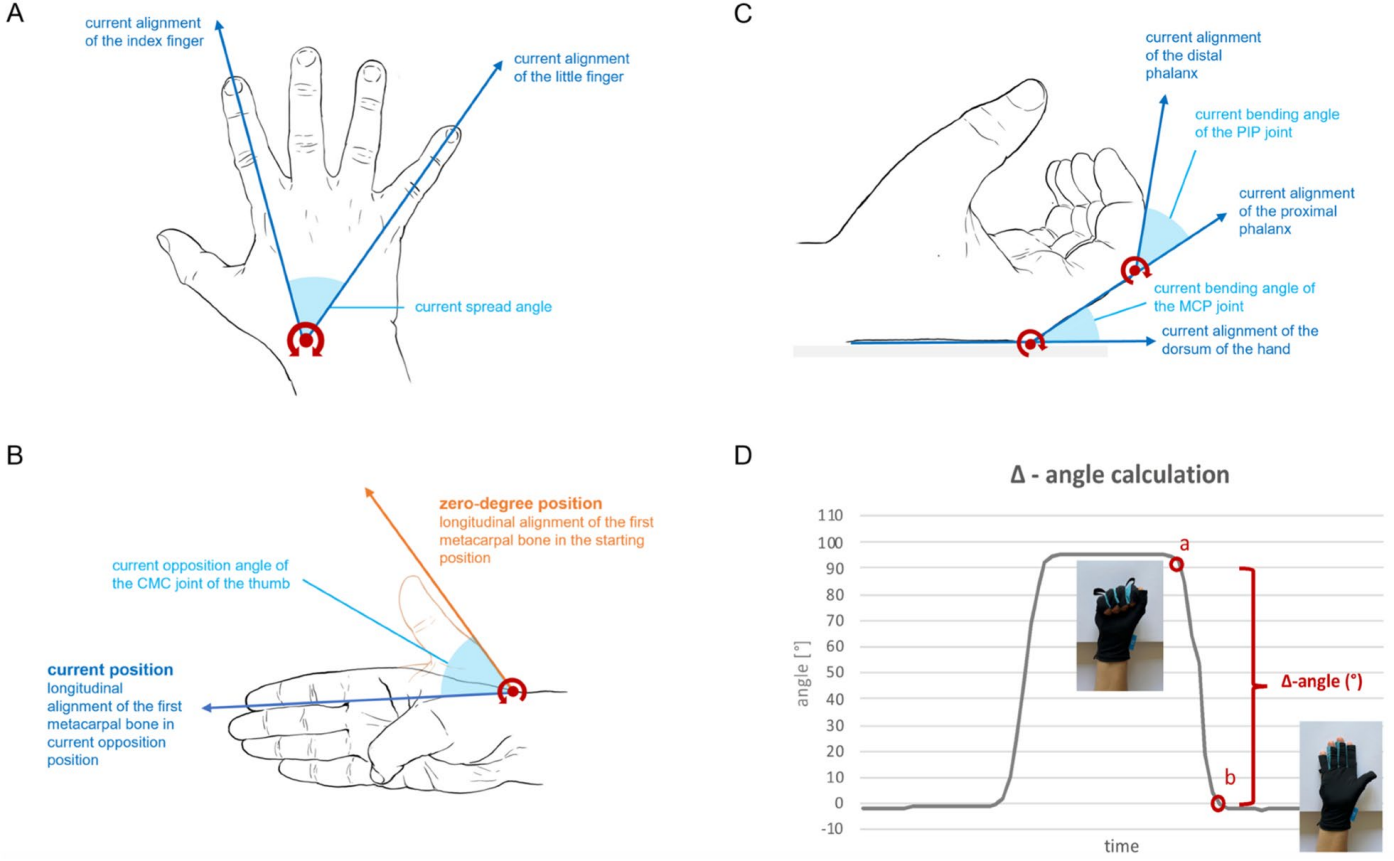
Angle measurement and analysis of ROM by the data glove. The current spread angle between the little and the index finger, measured during the *finger spread* movement pattern (light blue area), results from the current alignment of the index and the little finger (blue arrows) (**A**). The current bending angle of the metacarpophalangeal (MCP) joint and the proximal interphalangeal (PIP) joint of the long fingers (light blue areas), results from the alignment of the different sensors during the *fist opening* movement pattern (**B**). During the opposition movement of the thumb, the angle to of the carpometacarpal (CMC) joint was determined. The orange arrow signifies the longitudinal alignment of the sensor at the first metacarpal bone in the so-called *zero-degree position*). The blue arrow indicates the longitudinal alignment of the first metacarpal bone in the current opposition position. In between, the current opposition angle of the CMC joint is illustrated (light blue area). **C** The maximum range of motion (ROM) is calculated based on the time-angle signal, exemplarily shown for a single movement during the fist opening movement pattern. The maximum angle was measured when forming a fully clenched fist (a). The minimum angle was measured when the long fingers were fully extended (b). The difference between these two points (a, b) is referred to as the delta (Δ)—*angle* (in °) and represents the maximum ROM (D). The rotation axis is always shown in red

The specific angles, measured by the data glove during the three distinct movement patterns about 25 times per second, and their corresponding axes of rotation are depicted in Fig. 3 A-C. To determine the maximum ROM of the hand, we calculated the difference between the angle in the maximum and the minimum position (in °) for each of the alternating movements during glove assessment. In the following, this difference is referred to as the *Δ—angle* (Fig. 3D). As for each run through the course 50 obstacles had to be passed, half of them required a movement, which is essentially mediated by one of the three main hand nerves. In theory, this resulted in 25 Δ—angles for each glove assessment. The resulting Δ—angles were revised and their mean was the parameter of interest for all analyses concerning the glove (Additional file 1).

The corresponding data revision and processing software is described in Additional file 1.

### Patients’ satisfaction survey

One part of the feasibility assessment was a standardized questionnaire that evaluated whether and why the patients would (not) recommend the examinations with the data glove to others based on their individual experience in this study. Furthermore, we asked the patients whether they would prefer the data glove assessment to any of the routine outcome measures (Vigorimeter, NCS, HRUS, everyday-life questionnaires), and if so, why.

### Clinical outcome parameters and PROMs

We performed clinical examination at each time point and followed the current recommendations by using multidimensional array of established outcome measures [1, 5, 13]. To address the domain of *impairment*, the examination included the assessment of grip strength by the Martin Vigorimeter [16, 40]. The mean of three consecutive measurements was calculated [16]. Furthermore, we applied MRC Sum Score to examine muscle strength [18]. The *disability* domain was represented by both the INCAT disability score [17, 41] and the disease-specific R-ODS, quantified in log-odds (logits) [20, 25]. As the focus of this study was on the hand motor function of the patients, the respective arm sub-score was extracted from the MRC Sum Score and the INCAT disability score and used for the analyses. The *symptoms* domain was referred to by assessing the Beck Depression Inventory (BDI) [21] and the Fatigue Severity Scale (FSS) [22], which were not mainly focused on in this study.

### Nerve conduction studies

We performed NCS at T_2_ and T_4_ to assess nerve conduction velocity (NCV) of the three hand nerves (median–motor NCV, ulnar–motor NCV, and (superficial) radial nerve–sensory NCV) at the forearm. We therefore used standard neurophysiology devices (Natus Neurology, Nicolet EDX), and performed all studies with a surface stimulator and surface recording electrodes.

### High-resolution ultrasound

To assess the morphology of the peripheral nerves, we measured the cross-sectional area (CSA) of specific nerve sections at T_2_ and T_4_. We used a Mindray TE7 ultrasound scanner with a 3–13 MHz linear transducer. Maximum CSA of the median and ulnar nerve were measured at the upper arm and for the radial nerve at the radial sulcus.

NCS and HRUS were only conducted at two time points (T_2_ and T_4_) primarily due to time constraints and the associated burden on patients, which made it impractical to perform these assessments at every time point.

An overview of the assessments during the study course can be found in Table 1.

**Table 1:** Assessments during the study course

Time points	Measurements
T_0_/T_1_/T_3_	MRC (arm sub-score), INCAT disability (arm sub-score), Vigorimeter, R-ODS (logits), BDI, FSS
T_2_/T_4_	MRC (arm sub-score), INCAT disability (arm sub-score), Vigorimeter, R-ODS (logits), BDI, FSS, NCS, HRUS

MRC, Medical Research Council; INCAT, Inflammatory Neuropathy Cause and Treatment; R-ODS, Rasch-built Overall Disability Scale; BDI, Beck Depression Inventory; FSS, Fatigue Severity Scale; NCS, nerve conduction studies; HRUS, high-resolution ultrasound

### Statistical analyses

We conducted statistical analyses using RStudio (Version 4.2.2) and Graph Pad Prism (Version 9.5.1) on MacOS. The Shapiro–Wilk test, supported by Q-Q plots, assessed data normality. Data were reported as mean (SD) for normally distributed metrics and median (IQR) for ordinal or non-normal metrics. Data analysis included information up to the point of participant withdrawal for those lost to follow-up. The level of statistical significance was defined as α = 0.05.

Analyses focused solely on data from the dominant, affected side. To assess test–retest reliability, we calculated Intraclass correlation coefficients (ICCs) for metrically scaled parameters, using a two-way mixed-effects model for absolute agreement (guidelines by Koo and Li [42]). For ordinal data, we applied the linearly weighted Cohen’s Kappa, following Landis and Koch [43]. To compare the ROM of patients with and without relevant hand motor impairment, we applied unpaired, two-sided Student´s t-tests including Welch correction at T_2_. We performed receiver operating characteristics (ROC) analyses for the three glove movement patterns and ascertained the area under the curve (AUC), as well as a specific cut off value and the corresponding sensitivity and specificity. AUC was then rated according to the literature [44]. Additionally, we performed ROC analyses for the metrically scaled established outcome parameters (Vigorimeter, R-ODS). Finally, we did a composite ROC analysis comparing the performance of the glove movement patterns with that of NCV and CSA. We therefore calculated z-scores and applied the mean of each parameter.

For analyses targeting the data glove's ability to assess hand motor function, we only included patients with clinically relevant hand motor impairment. We assessed the construct validity of the data glove by analyzing correlations between the glove measurements and well-established clinical parameters at T_2_. These correlations were calculated to determine, how effectively the glove reflects hand motor function impairment. They were measured using Pearson’s correlation for normally distributed data and Kendall-Tau for ordinal or non-normal data, without Bonferroni correction for multiple testing. Correlation effect sizes were interpreted following Cohen [45]. Additional correlation analyses between glove movement patterns and NCV/CSA of hand nerves were detailed in Additional file 5. A Linear Mixed Model (LMM) with time as a fixed effect and person as a random effect handled longitudinal data, with more details in Additional file 3.

## Results

### Patients’ characteristics

25 patients with chronic immune-mediated neuropathies were included in this study. Despite one patient with hand motor impairment who missed T_4_ for personal reasons, all patients completed all follow-up time points of the study. Two patients did not complete the movement pattern finger spread at all time points, and one only at two of the time points due to insufficient ROM. One patient was excluded from the test–retest reliability analysis, because of deviation from the study protocol. Moreover, the NCV of the median nerve at the forearm was not assessed at T_2_ for one patient. The average follow-up time was 23.2 weeks, ranging from 10.8 weeks to 49.1 weeks.

Characteristics of the study cohort can be found in Table 2. Overall, 14 out of 25 patients were found to have clinically relevant hand motor impairment. Patients with relevant hand motor impairment performed (significantly) worse concerning all of the clinical outcome parameters and PROMs than patients without (Additional file 4). During the study course, treatment adaptations (i.e. dose reduction/interval lengthening) occurred for 18 patients (72.0%).

**Table 2:** Patients’ characteristics differentiated for the different subgroups

	Total (n = 25)	Patients with hand motor impairment (n = 14)	Patients without hand motor impairment (n = 11)
Demographics			
Gender distribution	: 5 : 20	: 4 : 10	: 1 : 10
Age (years)	64.24 (9.70) years	63.29 (10.04) years	66.45 (8.43) years
Height (m)	1.78 (0.11) m	1.73 (0.11) m	1.85 (0.07) m
Weight	92.10 (24.25) kg	82.36 (17.47) kg	104.51 (27.68) kg
Chronic immune-mediated neuropathy—subtypes	n = 25	n = 14	n = 11
CIDP	n = 23	n = 12	n = 11
Typical CIDP	n = 4	n = 4	n = 0
Multifocal CIDP	n = 11	n = 5	n = 6
Focal CIDP	n = 1	n = 1	n = 0
Distal CIDP	n = 3	n = 2	n = 1
Sensory-predominant CIDP	n = 4	n = 0	n = 4
MMN	n = 2	n = 2	n = 0
IVIg treatment regimen			
Mean dose per kg at the start of the study	0.87 (0.17) g/kg	0.88 (0.17) g/kg	0.85 (0.17) g/kg
Mean dose per kg at the end of the study	0.84 (0.18) g/kg	0.85 (0.20) g/kg	0.83 (0.17) g/kg
Mean interval length at the start of the study	5.36 (2.3) weeks	5.17 (2.43) weeks	5.61 (2.29) weeks
Mean interval length at the end of the study	6.34 (2.38) weeks	6.37 (2.51) weeks	6.30 (2.32) weeks
Treatment reduction	Yes: n = 18 No: n = 7	Yes: n = 11 No: n = 3	Yes: n = 7 No: n = 4
Dominant hand			
Right	n = 23	n = 12	n = 11
Left	n = 2	n = 2	n = 0

Metric and normally distributed data are listed as mean (standard deviation)

IVIg, intravenous immunoglobulin; CIDP, chronic inflammatory demyelinating polyneuropathy; MMN, multifocal motor neuropathy)

(Tab. 2), at the discretion of the treating physician according to established guidelines [4, 5].

### Test–retest reliability

Test–retest reliability analysis, based on the data of T_1_ and T_2_, revealed *excellent* (ICC) and *almost perfect* (Cohen´s Kappa) reliability coefficients for the four established clinical parameters (Vigorimeter, INCAT (arm sub-score), R-ODS and MRC (arm sub-score). (Table 3).

**Table 3:** Test–retest reliability coefficients of the clinical parameters

clinical parameter	ICC/Cohen’s Kappa result	95% CI	Rating	Included patients
Vigorimeter grip strength	0.98 (ICC)	0.96–0.99	excellent	n = 24
INCAT (arm sub-score)	0.87 (Cohen´s Kappa)	0.74–1.00	almost perfect	n = 24
MRC (arm sub-score)	0.84 (Cohen´s Kappa)	0.67–0.97	almost perfect	n = 24
R-ODS (logits)	0.94 (ICC)	0.87–0.98	excellent	n = 24

ICC and Cohen´s-Kappa results for T_1_—T_2_ were rated according to Koo and Li [42] and Landis and Koch [43]

ICC, Intraclass correlation coefficient; INCAT, Inflammatory Neuropathy Cause and Treatment; MRC, Medical Research and Treatment; RODS, Rasch-built Overall Disability Scale

The results of the Vigorimeter are shown for the dominant, affected hand

The results of the clinical outcome measures confirmed clinical stability (Table 3), providing the basis for an analysis of the test–retest reliability of the data glove. The ICC results of the glove movement patterns suggested *good* to *excellent* test–retest reliability (Table 4).

**Table 4:** Test–retest reliability coefficients of the data glove movement patterns

movement pattern of the glove	ICC/Cohen´s Kappa result	95% CI	Rating	Included patients
finger spread	0.94 (ICC)	0.82–0.98	Excellent	n = 21
thumb opposition	0.83 (ICC)	0.66–0.92	Good	n = 24
fist opening	0.87 (ICC)	0.73–0.94	Good	n = 24

ICC results were rated according to Koo and Li [42]

ICC, Intraclass correlation coefficient

The results are shown for the dominant, affected hand

Comparing ICC data from T_0_/T_1_ and T_1_/T_2_ revealed no relevant learning effect (data not shown).

### Detection of hand motor impairment

**Fig. 4:**
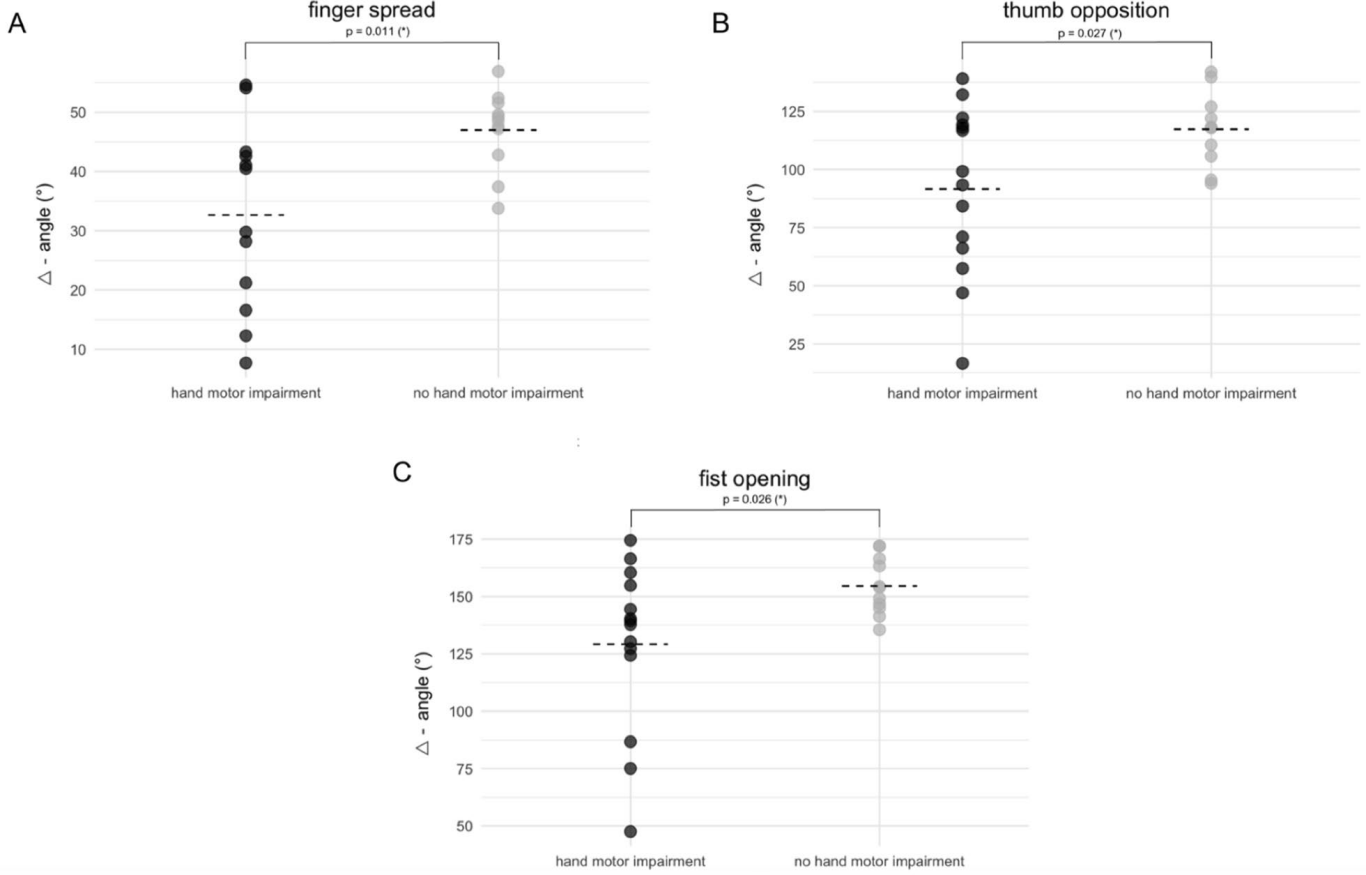
Comparison of the ROM between patients with and without clinically relevant hand motor impairment. Statistically significant differences were revealed for finger spread (**A**), thumb opposition (**B**) and fist opening (**C**) at T_2_ for the dominant, affected hand. Data are presented as individual data points. The mean of each group is indicated by the dotted lines. Statistical significance was tested using a Student’s t-test. (*p < 0.05; patients with hand impairment: n = 12 for the finger spread, n = 14 for the thumb opposition and the fist opening; patients without hand impairment: n = 14 for all glove movement patterns)

We found significant differences concerning the ROM of the fingers between patients with and without clinically relevant hand motor impairment (Fig. 4)*.* In this regard, the greatest difference between the two groups was demonstrated for the movement pattern finger spread, followed by thumb opposition and fist opening (finger spread: 32.7 (15.8)° vs. 47.0 (6.7)°, p = 0.011; thumb opposition: 91.6 (36.1)° vs. 117.3 (15.6)°, p = 0,027; fist opening: 129.2 (36.2)° vs. 154.5 (12.4)°, p = 0.026).

**Fig. 5:**
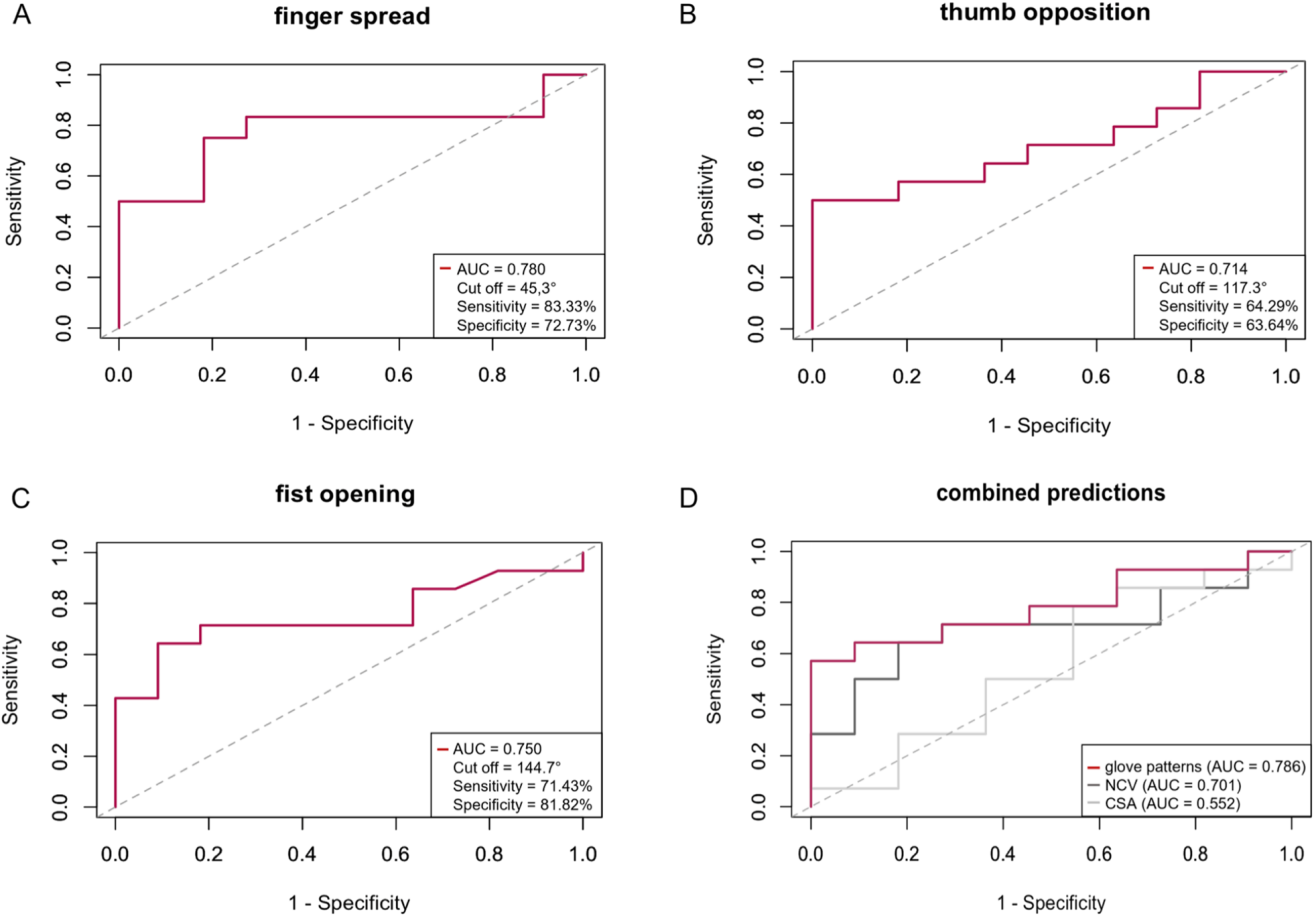
Results of the ROC analysis for the glove movement patterns and the NCV/CSA. The graphs A-C show the receiver operating characteristics (ROC) curve of the glove movement patterns finger spread (**A**), thumb opposition (**B**) and Fist opening (**C**) for the dominant, affected hand and the corresponding area under the curve (AUC). Furthermore, the chosen cut off value (in °) and the corresponding sensitivity and specificity are given. Graph D shows the combined predictions of the composite ROC analysis for the glove movement patterns, as well as for the nerve conduction velocity (NCV) and the cross-sectional area (CSA) as measured by ultrasound across the three main hand nerves (ulnar, median and superficial radial nerve). (n = 23 for the finger spread, n = 24 for the NCV of the median nerve, n = 25 for the thumb opposition, for the fist opening, for all CSA values, for the NCV of the ulnar and superficial radial nerve)

We assessed the diagnostic potential of the data glove to predict clinically relevant hand impairment by computing the AUC for each movement pattern. The discriminatory power of the different movement patterns to predict hand impairment can be rated as *fair* (AUC 0.714 – 0.780 Fig. 5) [44]. Overall, the composite ROC analysis revealed that the AUC of the glove movement patterns (AUC = 0.786) was still superior to that of established parameters such as NCV (AUC = 0.701) or ultrasound CSA measurement (AUC = 0.552) as shown by the combined predictions of each outcome measure (Fig. 5).

### Correlation analyses

In total, we calculated 12 correlations between four different established clinical parameters and the three glove movement patterns.

Correlation analyses of the clinical parameters at T_2_ showed a strong positive correlation between grip strength as recorded by the Vigorimeter and each of the three glove movement patterns (Table 5). Moreover, analyses of the INCAT (arm sub-score) revealed strong negative and significant correlations for the movement patterns finger spread and thumb opposition and moderate negative correlations for the fist opening movement pattern, although the latter was not statistically significant (Table 5). We found the strongest correlations for the thumb opposition (Table 5).

**Table 5:** Results of correlation analyses between the glove data and the clinical parameters

Movement pattern of the glove	Clinical parameter (impairment)			
	Vigorimeter grip strength		MRC (arm sub-score)	
	r	p-value	R	p-value
Finger spread	**0.64**	0.025 (*)	0.25	0.291
Thumb opposition	**0.77**	0.001 (**)	0.44	0.038 (*)
Fist opening	**0.62**	0.019 (*)	0.37	0.082

Movement pattern of the glove	Clinical parameter (disability)			
	INCAT (arm sub-score)		R-ODS (logits)	
	r	p-value	r	p-value
Finger spread	**− 0.56**	0.028 (*)	0.25	0.432
Thumb opposition	**− 0.66**	0.004 (**)	0.50	0.072
Fist opening	**− **0.41	0.073	0.35	0.225

Strong correlations are printed in bold

*p < 0.05, **p < 0.01; strong correlations are printed in bold, n = 12 for the finger spread movement pattern, n = 14 for the fist opening and the thumb opposition movement pattern

MRC, Medical Research; INCAT, Inflammatory Neuropathy Cause and Treatment; R-ODS, Rasch-built Overall Disability Scale

In contrast, besides the strong positive correlation between the R-ODS and the thumb opposition movement pattern, only moderate or weak positive correlations occurred between both the R-ODS and the MRC (arm sub-score) and each of the three data glove movement patterns (Table 5).

### Analyses of the longitudinal data

The repeated measures ANOVAs, also including multiple comparison analyses, did not reveal any significant differences for the glove movement patterns, Vigorimeter grip strength, or any of the other clinical parameters between any of the time points T_1_—T_4_, indicating clinical stability throughout the study course (Additional file 6).

Consistent with these findings, there was no evidence of a significant difference between the slope of the Vigorimeter as the reference tool and the slope of any of the three glove movement patterns (Additional file 6).

### Evaluation of the patients’ satisfaction survey

Most patients (79.4%) recommended the data glove based on their individual experience in this study—especially due to its relevance to everyday life, its ability to enhance motivation, and to regularly monitor the hand motor function in a standardized manner. In this context, more than half of the patients (52.0%) would prefer the data glove over NCS because they perceived the glove assessment to be much more comfortable.

## Discussion

This prospective proof-of-concept study evaluated a wearable data glove as an additional diagnostic and disease monitoring tool for patients with chronic immune-mediated neuropathies exhibiting significant hand motor impairment. To date, several data gloves, e.g., the NeuroAssess Glove [31], the Wü-Glove [34], or the SIGMA glove [46] have been investigated for measuring the motor dysfunction of the hands. Unlike previous data gloves, our glove assesses the function of all fingers simultaneously, offering comprehensive insights into hand motor function and overcoming the limitations of tools focused solely on individual finger movements [31, 37] or repetitive finger tapping [35, 36]. Moreover, the recent wearables were often primarily designed for rehabilitation [32, 38, 39].

To date, the use of data gloves in the context of chronic immune-mediated neuropathies has not been documented. Our glove differs from earlier models by providing a gamified environment to assess specific functions related to the three hand nerves, addressing the often patchy distribution of nerve involvement [4, 5]. This is why the three distinct glove movement patterns complement each other and should always be assessed and interpreted together to ensure the most valid and broad classification of hand motor impairment. The glove was shown to meet critical quality criteria for outcome measures, including construct validity based on moderate to strong correlations with established outcome measures and high patient acceptance due to its non-invasive nature (patients´ satisfaction survey). Essentially, it exhibited excellent test–retest reliability [25], comparable to established parameters like the R-ODS and the Vigorimeter and previous data glove approaches [31, 34, 47]. Furthermore, it demonstrated superior performance in certain movement patterns compared to traditional measures such as the MRC- and INCAT arm sub-scores.

The data of the glove were effectively interpreted using advanced statistical analyses, showing meaningful differences in hand motor function, with ROC analyses, based on our limited sample size, confirming sufficient though not remarkable sensitivity and specificity in identifying relevant impairments [44]. Notably, the finger spread movement provided the most accurate assessment, while the fist opening or thumb opposition movements were slightly less precise due to their complexity [48]. Additionally, the composite ROC analyses suggested advantages of the glove movement patterns over NCS and HRUS parameters. This is in line with previously published findings, highlighting the relatively poor suitability of NCS or nerve ultrasound as monitoring tools in chronic immune-mediated neuropathies (Additional file 5) [49, 50]. In general, the NCV and CSA values did not show pronounced deviations from the normative values, which could be attributed to the relatively long-standing immunomodulatory therapy that most patients have been undergoing [23, 24, 51]. In additional file 2, the *rocket game* and its features were presented. However, due to various confounding factors, the rocket game may not be as suitable for objective monitoring as the standardized game. Nevertheless, this game offers great potential for future rehabilitation applications, including integrated diagnostic approaches and home-based rehabilitation. The integration of gamification in the glove assessment aims to motivate patients to perform at their best within their capabilities, while making the process more enjoyable overall. By constructing exercises more interactive and engaging, motivation can be increased, and patients are encouraged to consistently participate in diagnostic but also in rehabilitative settings, even during repeated exercises. Gamification not only boosts engagement but also contributes to a more positive rehabilitation experience, as the patients gain direct feedback by the glove system. Based upon this, the glove could be potentially also used at home, with the software tracking and illustrating the progress patients have made over time.

As Dalakas pointed out, many patients tend to perceive subjective worsening, that cannot be sufficiently objectified, especially after treatment reduction or withdrawal [29]. Nevertheless, treatment decisions should be based on shared decision-making [13], which is essential for chronic diseases in general [28]. Sensor-equipped wearables such as the data glove evaluated in this study hold the promise to bridge the gap between subjective perception and objectively recorded data. If sufficient responsiveness will be shown, the glove could be offered to patients for occupational therapy or home use, allowing them to receive direct and objective feedback regarding the disease course. This could serve as reassurance, indicating that no significant deterioration has occurred by the time of their next medical appointment, and foster a trustful exchange between patients and physicians, especially in times of treatment withdrawal or -reduction.

Our results suggest the glove's potential for broader clinical application, not only within specialized centers but also in peripheral settings or during occupational therapy, as it is investigator independent. The three movement patterns assessed by the glove—thumb opposition, finger spread, and fist opening—are crucial for daily activities, for example with thumb opposition playing a key role in grasping objects like bottles [37]. Altogether, these movements encapsulate the primary hand functions mediated by the median, ulnar, and radial nerves, offering a comprehensive overview of a patient's motor hand function.

The glove's clinical value is highlighted by its advantages over traditional goniometry, which, while considered the gold standard, can be time-consuming and may lack accuracy [33]. Goniometers typically assess one joint at a time and capture only static measurements, limiting insights into dynamic motion [52]. In contrast, the goniometric glove facilitates simultaneous recording of multiple joint positions, significantly expediting the assessment process, while also reducing investigator dependency and enhancing consistency.

Furthermore, unlike traditional measures such as the MRC sum score, which often lack functional relevance, and subjective questionnaires like the R-ODS, the glove effectively integrates objective measurements with functional applicability. Our patients noted they had the impression that their impairment was accurately reflected by the glove, providing reassurance and confidence in the assessment process. The high acceptance and stability observed in patients highlight the glove's capability to monitor disease progression reliably.

A limitation of the glove is that especially the movement pattern finger spread cannot be validly used by patients with very severe impairment of hand motor function. This could also be applied to the fist opening and thumb opposition movement pattern. Similarly, the Jamar Dynamometer cannot be used by these patients either as it too requires a certain level of hand function, e.g., due to its weight [53].

Another limitation of the study lies in the unequal gender distribution, with more male than female patients. This may have caused minor, but not significant, variations concerning glove measurements due to the differences in hand size, particularly between males and females. The use of a single glove size led to slight divergences in sensor alignment, though the fit remained consistent for each patient over time. While more males are typically affected by CIDP [54], the imbalance was due to the necessity of recruiting all eligible patients, given the rarity of the disease. By now, a range of different glove sizes is available, allowing for better customization to individual hand sizes in future studies.

Future studies should explore its utility in detecting clinically meaningful changes, especially in treatment-naive patients or those undergoing significant therapy adjustments [36], as this would be a prerequisite to use the glove in guiding treatment decisions. Moreover, the inclusion of a healthy control group and the customization of individual glove sizes would enhance the generalizability of our findings. The measurement of the data glove is not exclusive to patients suffering from chronic immune-mediated neuropathies but could be also extended for rehabilitative purposes based on its gaming environment.

## Supplementary Information

Additional file 1. Explanation of the zero-degree position and specification of the corresponding data revision and processing software of the data glove. Explanation of the so-called zero-degree position determined during the Reset and step-by-step description of the data processing and the calculation of the final Δ—angles by the corresponding software, based on the time-angle signal.

Additional file 2. The rocket game. Introduction of the classical computer game, the *rocket game*, which should provide an outlook on potential applications of the data glove in the context of rehabilitation.

Additional file 3. Description of the LMM statistics. Illustration of the Linear Mixed Model design in R, – with and without an interaction term—adjustment for age and sex based on the literature. Description of various statistical analyses using LMM for different questionnaires, along with interpretation of the output in R.

Additional file 4. Means of the data glove movement patterns and the other outcome measures. Listed for the four relevant time points, differentiated for the subgroups of patients with and without clinically relevant hand motor impairment.

Additional file 5. Correlations between the data glove movement patterns and NCV/CSA. Description of the statistics and results of the correlations between the glove movement patterns and nerve conduction velocity at the forearm, respectively, the cross-sectional area at the upper arm of the corresponding nerves.

Additional file 6. Results of the longitudinal data analyses using LMM. Results of the ANOVA for the different clinical outcome measures and the glove movement patterns. P-values of the analyses using the LMM with an interaction term—comparing the slopes of the Vigorimeter and the three different glove movement patterns throughout the study.

## Data Availability

Anonymized datasets used in this study are available from the corresponding author on reasonable request. The corresponding data analysis software for the data glove can be found at Zenodo: https://zenodo.org/records/11619212. The software is made available under the Creative commons attribution 4.0 International license. Its requirements and further description can be found in Additional file 1.

## Abbreviations

ANOVA: Analysis of variance
AUC: Area under the curve
BDI: Beck Depression Inventory
CE: Conformité Européenne
CIDP: Chronic inflammatory demyelinating polyneuropathy
CSA: Cross-sectional area
CTS: Carpal tunnel syndrome
EFNS/PNS: European Federation of Neurological Societies/ Peripheral Nerve Society
FSS: Fatigue Severity Scale
Fig.: Figure
HRUS: High-resolution ultrasound
ICC: Intraclass correlation coefficient
INCAT: Inflammatory Neuropathy Cause and Treatment
IQR: Interquartile range
IVIg: Intravenous immunoglobulin
LMM: Linear Mixed Model
log-odds: Logits
MCP: Metacarpophalangeal
MMN: Multifocal motor neuropathy
MRC: Medical Research Council
MS: Multiple sclerosis
NCS: Nerve conduction studies
NCV: Nerve conduction velocity
PIP: Proximal interphalangeal
PROM: Patient-reported outcome measure
QoL: Quality of Life
ROM: Range of motion
ROC: Receiver operating characteristics
R-ODS: Rasch-built Overall Disability Scale
SD: Standard deviation
Tab.: Table

## References

[1] Allen JA, Eftimov F, Querol L. Outcome measures and biomarkers in chronic inflammatory demyelinating polyradiculoneuropathy: from research to clinical practice. Expert Rev Neurother. 2021;21(7):805–16. 34130574 10.1080/14737175.2021.1944104

[2] Kieseier BC, Mathey EK, Sommer C, Hartung HP. Immune-mediated neuropathies. Nat Rev Dis Primers. 2018;4(1):31. 30310069 10.1038/s41572-018-0027-2

[3] Goedee HS, Rajabally YA. Evidence base for investigative and therapeutic modalities in chronic inflammatory demyelinating polyneuropathy and multifocal motor neuropathy. Neurodegener Dis Manag. 2022;12(1):35–47. 35007438 10.2217/nmt-2021-0015

[4] Joint Task Force of the Efns and the PNS. European Federation of Neurological Societies/Peripheral Nerve Society guideline on management of multifocal motor neuropathy. Report of a joint task force of the European Federation of Neurological Societies and the Peripheral Nerve Society–first revision. J Peripher Nerv Syst. 2010;15(4):295–301. 21199100 10.1111/j.1529-8027.2010.00290.x

[5] Van den Bergh PYK, van Doorn PA, Hadden RDM, Avau B, Vankrunkelsven P, Allen JA, et al. European academy of neurology/Peripheral nerve society guideline on diagnosis and treatment of chronic inflammatory demyelinating polyradiculoneuropathy: report of a joint Task Force-Second revision. J Peripher Nerv Syst. 2021;26(3):242–68. 34085743 10.1111/jns.12455

[6] Adrichem ME, Lucke IM, Vrancken A, Goedee HS, Wieske L, Dijkgraaf MGW, et al. Withdrawal of intravenous immunoglobulin in chronic inflammatory demyelinating polyradiculoneuropathy. Brain. 2022;145(5):1641–52. 35139161 10.1093/brain/awac054 PMC9166547

[7] Mengel D, Fraune L, Sommer N, Stettner M, Reese JP, Dams J, et al. Costs of illness in chronic inflammatory demyelinating polyneuropathy in Germany. Muscle Nerve. 2018;58(5):681–7. 30073683 10.1002/mus.26315

[8] Stino AM, Naddaf E, Dyck PJ, Dyck PJB. Chronic inflammatory demyelinating polyradiculoneuropathy-diagnostic pitfalls and treatment approach. Muscle Nerve. 2021;63(2):157–69. 32914902 10.1002/mus.27046

[9] Vlam L, Cats EA, Willemse E, Franssen H, Medic J, Piepers S, et al. Pharmacokinetics of intravenous immunoglobulin in multifocal motor neuropathy. J Neurol Neurosurg Psychiatry. 2014;85(10):1145–8. 24336791 10.1136/jnnp-2013-306227

[10] Fokkink W, Koch B, Ramakers C, van Doorn PA, van Gelder T, Jacobs BC. Pharmacokinetics and pharmacodynamics of intravenous immunoglobulin G Maintenance Therapy in Chronic Immune-mediated Neuropathies. Clin Pharmacol Ther. 2017;102(4):709–16. 28378901 10.1002/cpt.693

[11] Jovanovich E, Karam C. Human immune globulin infusion in the management of multifocal motor neuropathy. Degener Neurol Neuromuscul Dis. 2016;6:1–12. 30050363 10.2147/DNND.S96258 PMC6053084

[12] Allen JA, Berger M, Querol L, Kuitwaard K, Hadden RD. Individualized immunoglobulin therapy in chronic immune-mediated peripheral neuropathies. J Peripher Nerv Syst. 2018;23(2):78–87. 29573033 10.1111/jns.12262 PMC6033159

[13] Allen JA, Merkies ISJ, Lewis RA. Monitoring clinical course and treatment response in chronic inflammatory demyelinating polyneuropathy during routine care: a review of clinical and laboratory assessment measures. JAMA Neurol. 2020;77(9):1159–66. 32338716 10.1001/jamaneurol.2020.0781

[14] Vanhoutte EK, Faber CG, Merkies IS, PeriNom SS. 196th ENMC international workshop: outcome measures in inflammatory peripheral neuropathies 8–10 February 2013, Naarden, The Netherlands. Neuromuscul Disord. 2013;23(11):924–33. 23835324 10.1016/j.nmd.2013.06.006

[15] Pruppers MH, Draak TH, Vanhoutte EK, Van der Pol WL, Gorson KC, Leger JM, et al. Outcome measures in MMN revisited: further improvement needed. J Peripher Nerv Syst. 2015;20(3):306–18. 26115442 10.1111/jns.12124

[16] Merkies IS, Schmitz PI, Samijn JP, Meche FG, Toyka KV, van Doorn PA. Assessing grip strength in healthy individuals and patients with immune-mediated polyneuropathies. Muscle Nerve. 2000;23(9):1393–401. 10951442 10.1002/1097-4598(200009)23:9<1393::aid-mus10>3.0.co;2-o

[17] Hughes R, Bensa S, Willison H, Van den Bergh P, Comi G, Illa I, et al. Randomized controlled trial of intravenous immunoglobulin versus oral prednisolone in chronic inflammatory demyelinating polyradiculoneuropathy. Ann Neurol. 2001;50(2):195–201. 11506402 10.1002/ana.1088

[18] Compston A. Aids to the investigation of peripheral nerve injuries. Medical Research Council: Nerve Injuries Research Committee. His Majesty’s Stationery Office: 1942; pp. 48 (iii) and 74 figures and 7 diagrams; with aids to the examination of the peripheral nervous system. By Michael O’Brien for the Guarantors of Brain. Saunders Elsevier: 2010; pp. [8] 64 and 94 Figures. Brain. 2010;133(10):2838–44. 20928945 10.1093/brain/awq270

[19] van Nes SI, Vanhoutte EK, van Doorn PA, Hermans M, Bakkers M, Kuitwaard K, et al. Rasch-built overall disability scale (R-ODS) for immune-mediated peripheral neuropathies. Neurology. 2011;76(4):337–45. 21263135 10.1212/WNL.0b013e318208824b

[20] Vanhoutte EK, Faber CG, van Nes SI, Cats EA, Van der Pol WL, Gorson KC, et al. Rasch-built overall disability scale for multifocal motor neuropathy (MMN-RODS((c)) ). J Peripher Nerv Syst. 2015;20(3):296–305. 26329270 10.1111/jns.12141

[21] Wang YP, Gorenstein C. Psychometric properties of the beck depression inventory-II: a comprehensive review. Braz J Psychiatry. 2013;35(4):416–31. 24402217 10.1590/1516-4446-2012-1048

[22] Valko PO, Bassetti CL, Bloch KE, Held U, Baumann CR. Validation of the fatigue severity scale in a Swiss cohort. Sleep. 2008;31(11):1601–7. 19014080 10.1093/sleep/31.11.1601 PMC2579971

[23] Rattay TW, Winter N, Decard BF, Dammeier NM, Hartig F, Ceanga M, et al. Nerve ultrasound as follow-up tool in treated multifocal motor neuropathy. Eur J Neurol. 2017;24(9):1125–34. 28681489 10.1111/ene.13344

[24] Kerasnoudis A, Pitarokoili K, Gold R, Yoon MS. Nerve ultrasound and electrophysiology for therapy monitoring in chronic inflammatory demyelinating polyneuropathy. J Neuroimaging. 2015;25(6):931–9. 26242571 10.1111/jon.12279

[25] van Nes SI, Faber CG, Merkies IS. Outcome measures in immune-mediated neuropathies: the need to standardize their use and to understand the clinimetric essentials. J Peripher Nerv Syst. 2008;13(2):136–47. 18601658 10.1111/j.1529-8027.2008.00169.x

[26] Jerath NU, Strader SB, Reddy CG, Swenson A, Kimura J, Aul E. Factors influencing aversion to specific electrodiagnostic studies. Brain Behav. 2014;4(5):698–702. 25328846 10.1002/brb3.240 PMC4188363

[27] Rajabally YA, Fatehi F. Outcome measures for chronic inflammatory demyelinating polyneuropathy in research: relevance and applicability to clinical practice. Neurodegener Dis Manag. 2019;9(5):259–66. 31580223 10.2217/nmt-2019-0009

[28] Desroches S. Shared decision making and chronic diseases. Allergy Asthma Clin Immunol. 2010;6(Suppl 4):A8.

[29] Dalakas MC. Update on intravenous immunoglobulin in neurology: modulating neuro-autoimmunity, evolving factors on efficacy and dosing and challenges on stopping chronic IVIg therapy. Neurotherapeutics. 2021;18(4):2397–418. 34766257 10.1007/s13311-021-01108-4 PMC8585501

[30] Boukhris S, Magy L, Gallouedec G, Khalil M, Couratier P, Gil J, et al. Fatigue as the main presenting symptom of chronic inflammatory demyelinating polyradiculoneuropathy: a study of 11 cases. J Peripher Nerv Syst. 2005;10(3):329–37. 16221292 10.1111/j.1085-9489.2005.10311.x

[31] Oess NP, Wanek J, Curt A. Design and evaluation of a low-cost instrumented glove for hand function assessment. J Neuroeng Rehabil. 2012;9:2. 22248160 10.1186/1743-0003-9-2 PMC3305482

[32] Wang Q, Markopoulos P, Yu B, Chen W, Timmermans A. Interactive wearable systems for upper body rehabilitation: a systematic review. J Neuroeng Rehabil. 2017;14(1):20. 28284228 10.1186/s12984-017-0229-y PMC5346195

[33] Mohan A, Tharion G, Kumar RK, Devasahayam SR. An instrumented glove for monitoring hand function. Rev Sci Instrum. 2018;89(10): 105001. 30399736 10.1063/1.5038601

[34] Gentner R, Classen J. Development and evaluation of a low-cost sensor glove for assessment of human finger movements in neurophysiological settings. J Neurosci Methods. 2009;178(1):138–47. 19056422 10.1016/j.jneumeth.2008.11.005

[35] Alberti MA, Mori L, Francini L, Poggi I, Monti Bragadin M, Bellone E, et al. Innovative quantitative testing of hand function in Charcot-Marie-Tooth neuropathy. J Peripher Nerv Syst. 2015;20(4):410–4. 26456943 10.1111/jns.12150

[36] Carmisciano L, Signori A, Pardini M, Novi G, Lapucci C, Nesi L, et al. Assessing upper limb function in multiple sclerosis using an engineered glove. Eur J Neurol. 2020;27(12):2561–7. 32805743 10.1111/ene.14482

[37] Kuroiwa T, Fujita K, Nimura A, Miyamoto T, Sasaki T, Okawa A. A new method of measuring the thumb pronation and palmar abduction angles during opposition movement using a three-axis gyroscope. J Orthop Surg Res. 2018;13(1):288. 30445972 10.1186/s13018-018-0999-3 PMC6240257

[38] Golomb MR, McDonald BC, Warden SJ, Yonkman J, Saykin AJ, Shirley B, et al. In-home virtual reality videogame telerehabilitation in adolescents with hemiplegic cerebral palsy. Arch Phys Med Rehabil. 2010;91(1):1-8 e1. 20103390 10.1016/j.apmr.2009.08.153

[39] Park YS, An CS, Lim CG. Effects of a rehabilitation program using a wearable device on the upper limb function, performance of activities of daily living, and rehabilitation participation in patients with acute stroke. Int J Environ Res Public Health. 2021;18(11):5524. 34063970 10.3390/ijerph18115524 PMC8196786

[40] Desrosiers J, Hebert R, Bravo G, Dutil E. Comparison of the Jamar dynamometer and the Martin vigorimeter for grip strength measurements in a healthy elderly population. Scand J Rehabil Med. 1995;27(3):137–43. 8602475

[41] Breiner A, Barnett C, Bril V. INCAT disability score: a critical analysis of its measurement properties. Muscle Nerve. 2014;50(2):164–9. 24723454 10.1002/mus.24207

[42] Koo TK, Li MY. A guideline of selecting and reporting intraclass correlation coefficients for reliability research. J Chiropr Med. 2016;15(2):155–63. 27330520 10.1016/j.jcm.2016.02.012 PMC4913118

[43] Landis JR, Koch GG. The measurement of observer agreement for categorical data. Biometrics. 1977;33(1):159–74. 843571

[44] Ludemann L, Grieger W, Wurm R, Wust P, Zimmer C. Glioma assessment using quantitative blood volume maps generated by T1-weighted dynamic contrast-enhanced magnetic resonance imaging: a receiver operating characteristic study. Acta Radiol. 2006;47(3):303–10. 16613313 10.1080/02841850500539033

[45] Cohen J. A power primer. Psychol Bull. 1992;112(1):155–9. 19565683 10.1037//0033-2909.112.1.155

[46] Williams NW, Penrose JM, Caddy CM, Barnes E, Hose DR, Harley P. A goniometric glove for clinical hand assessment. Construction, calibration and validation. J Hand Surg Br. 2000;25(2):200–7. 11062583 10.1054/jhsb.1999.0360

[47] Mentzel M, Hofmann F, Ebinger T, Jatzold B, Kinzl L, Wachter NJ. Reproducibility of measuring the finger joint angle with a sensory glove. Handchir Mikrochir Plast Chir. 2001;33(1):59–63. 11258036 10.1055/s-2001-12082

[48] Jones LA, Lederman SJ. Human hand function. Oxford: Oxford University Press; 2006.

[49] Kerasnoudis A, Pitarokoili K, Behrendt V, Gold R, Yoon MS. Correlation of nerve ultrasound, electrophysiological and clinical findings in chronic inflammatory demyelinating polyneuropathy. J Neuroimaging. 2015;25(2):207–16. 24593005 10.1111/jon.12079

[50] Kerasnoudis A, Pitarokoili K, Behrendt V, Gold R, Yoon MS. Multifocal motor neuropathy: correlation of nerve ultrasound, electrophysiological, and clinical findings. J Peripher Nerv Syst. 2014;19(2):165–74. 24862982 10.1111/jns5.12067

[51] Khomand P, Katzberg H, Ngo M, Bril V. Electrophysiological responsiveness to long-term therapy in chronic inflammatory demyelinating polyneuropathy: case report. Case Rep Neurol. 2020;12(1):40–4. 32095131 10.1159/000505234 PMC7011741

[52] Wang L, Meydan T, Williams PI. A two-axis goniometric sensor for tracking finger motion. Sensors (Basel). 2017;17(4):770. 28379170 10.3390/s17040770 PMC5422043

[53] Draak TH, Pruppers MH, van Nes SI, Vanhoutte EK, Bakkers M, Gorson KC, et al. Grip strength comparison in immune-mediated neuropathies: vigorimeter vs. Jamar. J Peripher Nerv Syst. 2015;20(3):269–76. 26115516 10.1111/jns.12126

[54] Broers MC, Bunschoten C, Nieboer D, Lingsma HF, Jacobs BC. Incidence and prevalence of chronic inflammatory demyelinating polyradiculoneuropathy: a systematic review and meta-analysis. Neuroepidemiology. 2019;52(3–4):161–72. 30669140 10.1159/000494291 PMC6518865

