# Supplementary material for: Assessing hand motor function in chronic immune-mediated neuropathies: a proof-of-concept study using a data glove
Source: J Neuroeng Rehabil. 2024 Dec 20;21:218. doi: 10.1186/s12984-024-01518-3 (PMC11662497; doi:10.1186/s12984-024-01518-3)
Supplement: Supplementary file 1 — Additional file 1. Explanation of the zero-degree position and specification of the corresponding data revision and processing software of the data glove. Explanation of the so-called zero-degree position determined during the Reset and step-by-step description of the data processing and the calculation of the final Δ—angles by the corresponding software, based on the time-angle signal. [file 12984_2024_1518_MOESM1_ESM.docx]

**Additional file 1 – Explanation of the zero-degree position and specification of the corresponding data processing and analysis software of the data glove**

**Zero-degree position of the movement patterns**

The so-called *zero-degree position* served as the reference point for the angle determination of one run through the course. It was determined during the *Reset* before each glove assessment and served as the baseline of the time-angle signal (zero degree in Fig. 3D). For the fist opening, this corresponded to the position of fully flexed long fingers (right image of Fig. 2C), and for the finger spread, it corresponded to the position where the long fingers were maximally brought together (left image of Fig. 2A). Finally, for the thumb opposition we defined the zero-degree position as the position in which the thumb is maximally abducted (left image Fig. 2B and orange arrow in Fig. 3C).

**Specification of the corresponding data processing and analysis software of the data glove**

Raw data was revised and analyzed using an algorithm developed specifically for this study. It ensures the objectivity of the glove assessment based on a standardized process. The data analysis software was written in Python, utilizing the *numpy* and *pandas* libraries. Python 3.10.5 was used for developing and running the program. This software is made available under the Creative Commons Attribution 4.0 International license and can be found at Zenodo (https://zenodo.org/records/10318488) and further details are provided below:

All three glove movement patterns were assessed once at each of the five consecutive timepoints, resulting in five distinct data sets for the finger spread, five for the thumb opposition and five for the fist opening movement pattern for every patient. Each data set was analyzed separately.

In the initial step, the relevant angles were determined corresponding to the respective movement pattern. For the *finger spread* movement pattern, the spread angle between the index and little fingers was calculated. In the case of *thumb opposition,* the opposition angle of the carpometacarpal (CMC) joint and the bending angle of the metacarpophalangeal (MCP) joint were combined. For the *fist opening* movement pattern, the bending angle of the MCP and the PIP joint was summed up for the individual long fingers, resulting in a distinct data set for each of the long fingers.

Due to the nature of the alternating movements during the data glove assessment, the underlying time-angle signal could be approximated using a *square wave* to determine the minimum and maximum angle of each alternating movement. Sometimes irregularities such as spikes or smaller intermediate motions could appear in the data set. These could have been caused by momentary lapses in concentration or twitching. To precisely reflect the actual range of motion of the patients, these irregularities should be ignored. Thus, the signal was revised in the following way:

First, data spikes (rapid changes in the signal within a narrow time frame) were identified and removed. Next, a *moving average* was used to classify the maxima and minima of the square wave signal. If the time-angle signal fell below the moving average, the values were relevant for determining a minimum. In contrast to this, if the time-angle signal exceeded the moving average, the values were relevant for determining a maximum.

If the aforementioned involuntary twitches or motions occurred, such patterns were detected and disregarded by the algorithm, focusing only on the actual start- and endpoint of a movement. For this purpose, the program could combine two movements into one, if this seemed to be the better interpretation. The process was repeated until there was no need for further revision.

Additionally, the signal was classified into the categories of *good, moderate,* and *poor* based on the extent of data noise that needed to be filtered. If the quality was classified as poor, a manual evaluation of the movement pattern was performed.

**
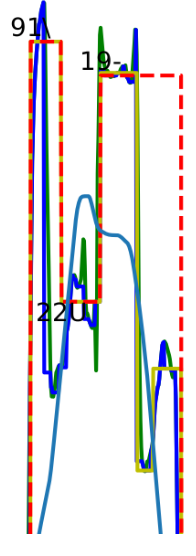
**

**Additional file 1 – Figure 1: Combining bars of involuntary twitches or motions.** This graphic shows an example of a movement including involuntary motions, as the time-angle signal does not go straight down but is interrupted. In the first iteration, the program identified three bars. The light green line contains an unusual movement: It goes down pretty far, then a little bit up, and then far down again. These types of patterns are recognized by the program as an indicator that the two recognized movements should be considered as only one movement (i.e., involuntary twitches and motions). The corresponding bars are then combined into one (dashed red line).

In the following, the Δ - angles were calculated for each assessment of a specific glove movement pattern as the difference between each minimum and maximum of the revised time-angle signal. This was only done for the movement direction, which is mainly mediated by one of the three main hand nerves, as described in the methods section (Figure 1D).


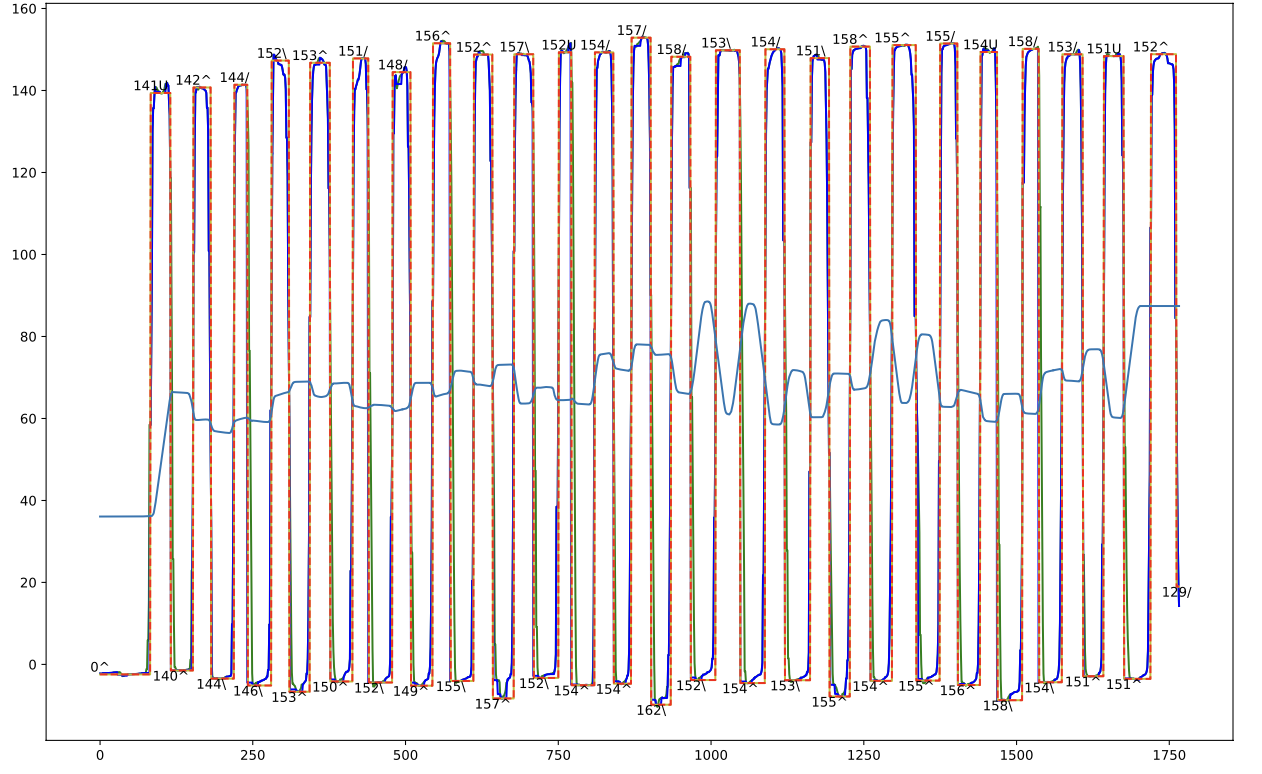


30
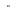


10

0

20

40
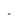

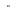


50
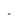

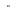


60
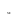

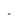

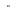


70
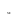

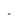

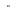


**Additional file 1 – Figure 2: The raw time-angle signal and its approximation by a square wave signal**. This graphic exemplarily shows the data of one run through the course of the fist opening movement pattern and its alternating movements. The raw time-angle signal is marked in dark blue and the approximation by the square wave as the dashed red line. On the X-axis, time is represented (in s) and on the Y-axis, the angle measured by the data glove is depicted (in °). The light blue line represents the moving average, which was the basis of the maxima and minima classification. At the peaks, the difference between the maximum and the minimum angle is presented. For the fist opening, the corresponding Δ - angle (in °) can be found at the minima.

Out of the calculated Δ - angles of one run through the course, only the maximum *n* angles were taken into account, where *n* was the number of movements a patient had to perform at a minimum to complete the course of the alternating obstacles. Moreover, the first Δ - angle was excluded from the analysis, as it was often invalid.

In the next step, outliers among the maximum *n* Δ - angles were identified and excluded using the 1,5*interquartile range (IQR) method.

Subsequently, the *mean* of the remaining Δ - angles was calculated. For the fist opening movement pattern, the means of the Δ - angles of the four individual long fingers were averaged, to have one comparable value per movement pattern.

This mean was the parameter of interest for the subsequent statistical analyses, because we aimed for a comprehensive representation of the ROM and robust statistical analyses. Thereby, we intended to minimize the impact of confounding factors, such as temporary inattention, lack of compliance or fatigue towards the end of a glove performance.
