# Supplementary material for: Assessing hand motor function in chronic immune-mediated neuropathies: a proof-of-concept study using a data glove
Source: J Neuroeng Rehabil. 2024 Dec 20;21:218. doi: 10.1186/s12984-024-01518-3 (PMC11662497; doi:10.1186/s12984-024-01518-3)
Supplement: Supplementary file 2 — Additional file 2. The rocket game. Introduction of the classical computer game, the rocket game, which should provide an outlook on potential applications of the data glove in the context of rehabilitation. [file 12984_2024_1518_MOESM2_ESM.docx]

**Additional file 2 – The rocket game**

A classic computer game was integrated into this study to emphasize the interactive gaming character of the data glove and its associated software. The game, known as the rocket game, involved guiding an animated rocket based on simultaneous recording of raw values from associated movement exercises. Each study session concluded with the rocket game.
The patients were asked to collect as many points as possible by catching stars. A total score was generated based on distance traveled and stars collected. The height of the rocket was controlled by opening and closing the fist: clenching the fist caused the rocket to rise while opening it caused it to drop. This exercise was performed in the same manner as the *fist opening* movement pattern.

The rocket crashed upon hitting the outer edge of the playing field. In this case, the game restarted, but time continued to run down, and the points already scored were retained.
A standardized playing time of two minutes was set for the *rocket game*. The rocket's flight speed increased throughout the game, depending on how many stars have been captured and the resulting score. Overall, the game was divided into six different levels of difficulty, which could be completed within the game. A higher difficulty level was characterized by faster rocket speed as well as a hilly profile of the upper and lower edges of the playing field. Speed was reduced by one difficulty level after a crash.


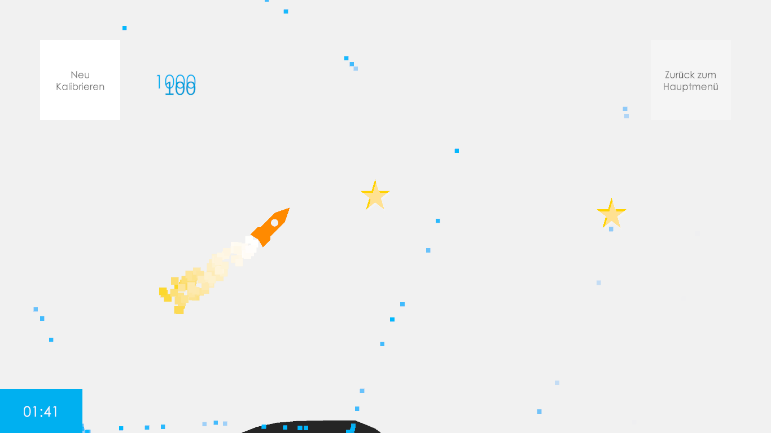


**Additional file 2 - Figure 1: Rocket game visualization.** This screenshot shows the above-mentioned *rocket game*. The patients should collect the stars visible here by controlling the rocket using the balling or opening of the fist, exactly as for the fist opening movement pattern of the glove. They should generate as many points as possible within the standardized time frame of two minutes by collecting the stars as described.

The longitudinal course of the rocket game score fluctuated relevantly between the different time points. Consequently, this parameter proved to be less stable over time compared to all established measurement instruments, as well as all three glove movement patterns. This could be due to learning effects, variations in daily form, differences in concentration, motivation or fatigue.
A ROC analysis of the scores at T_2_ revealed an AUC of 0.614. This could be possibly explained, among other factors, by a difference in familiarity and previous experience with video games.
