# Supplementary material for: Assessing hand motor function in chronic immune-mediated neuropathies: a proof-of-concept study using a data glove
Source: J Neuroeng Rehabil. 2024 Dec 20;21:218. doi: 10.1186/s12984-024-01518-3 (PMC11662497; doi:10.1186/s12984-024-01518-3)
Supplement: Supplementary file 3 — Additional file 3. Description of the LMM statistics. Illustration of the Linear Mixed Model design in R, – with and without an interaction term—adjustment for age and sex based on the literature. Description of various statistical analyses using LMM for different questionnaires, along with interpretation of the output in R. [file 12984_2024_1518_MOESM3_ESM.docx]

**Additional file 3 – Description of the LMM statistics**

**1) Repeated measures Type III Analysis of Variance (ANOVA) with multiple comparisons**

To assess whether the patients revealed significant changes in the movement patterns and the clinical outcome measures throughout the course of the study, repeated measures Analyses of Variance (ANOVAs) using the *Satterthwaite* method were conducted. For this purpose, a Linear Mixed Model (LMM) was designed with *time* as a fixed effect and *person* as a random effect (random intercept). According to the literature, the LMM was further adjusted for age and sex [1-6].

The equation applied for these analyses was the following:

***randominterceptmodel <- lmer (outcome ~ timepoint + age + sex + (1/person), data = tool_data, REML=F)***

This equation was applied to the different outcome measures: the three glove movement patterns, the Vigorimeter as the primary clinical reference tool, as well as the R-ODS (logits), the INCAT (arm sub-score) and the MRC (arm sub-score). Multiple comparisons included *tukey adjustment.*

Absolute data was utilized for these analyses, *time* was defined as a categorial factor, referred to as *time point* in the LMM. The packages *lme4, lmerTest, and emmeans* were employed for this purpose.

**Example of the R code and the output using the example of the glove movement pattern *thumb opposition*:**

We checked whether the overall p-value for the variable *time point* in the Type III repeated measures ANOVA and for the multiple comparisons between the different time points was below 0.05. This was done to investigate if significant differences occurred in the various parameters throughout the study. If no significant differences were observed, we concluded that the patients revealed clinical stability concerning the tested outcome measures.

**2) Comparison of the recorded trends of the data glove movement patterns and the Vigorimeter using an LMM with an interaction term:**

The LMM was revised by including an interaction term: *timepoint*tool* to investigate whether the trend of the glove movement patterns differed significantly from the trend of the Vigorimeter throughout the study course. The Vigorimeter was chosen as the clinical reference tool as it is known to be both reliable and responsive if meaningful changes occur [7, 8].

***randominterceptmodel <- lmer(outcome ~ timepoint * tool + age + sex + (1*/*person), data = glovemovementpattern_and_Vigorimeter_standardizeddata, REML=F)***

The *time*, again referred to as *time point* in the LMM, was modeled as a continuous and z-scores (standardized data) were applied to ensure comparability between the two tools despite the different dimensions and units. The packages used for this analysis included lme*4, lmerTest, lmerperformance,* and *lmerreghelper.*

**Example of the R Code and the output using the example of the glove movement pattern *thumb opposition:***

**
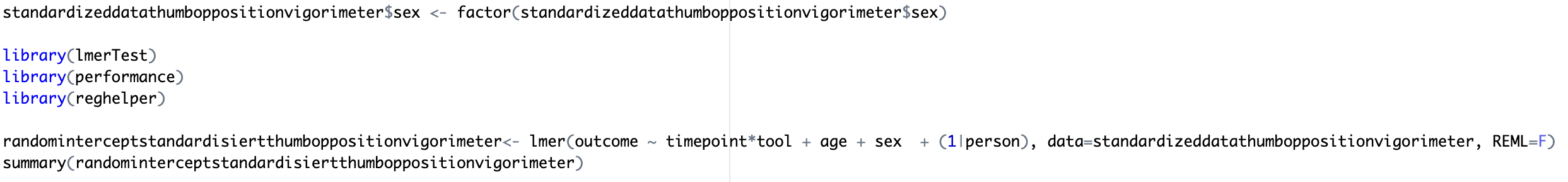
**


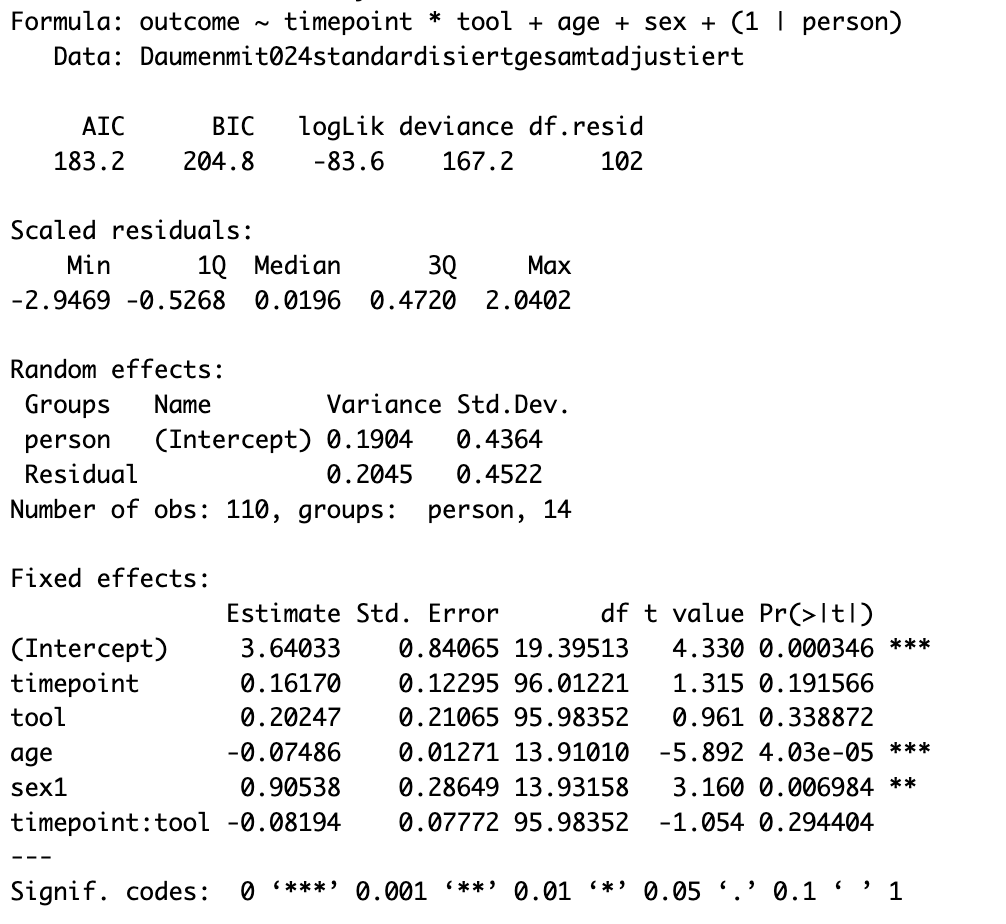


To assess whether the relationship between the independent variable *time point* and the dependent variable *outcome* was determined by the expression of the independent variable *tool* (either glove movement pattern or Vigorimeter), we checked if the p-value of the interaction term was less than 0.05. A p-value < 0.05 would indicate a significant interaction between the two independent variables *time point* and *tool*, thereby signifying a significant difference between the slopes of the data glove´s movement patterns and the Vigorimeter.

**References**

1. Smahel Z, Klimova A. The influence of age and exercise on the mobility of hand joints: 3. thumb joints. Acta Chir Plast. 2005;47(2):47-50.

2. Smahel Z, Klimova A. The effect of age and exercise on wrist mobility. Acta Chir Plast. 2005;47(3):92-7.

3. Yoshida R, House HO, Patterson RM, Shah MA, Viegas SF. Motion and morphology of the thumb metacarpophalangeal joint. J Hand Surg Am. 2003;28(5):753-7.

4. Bell RD, Hoshizaki TB. Relationships of age and sex with range of motion of seventeen joint actions in humans. Can J Appl Sport Sci. 1981;6(4):202-6.

5. McKay MJ, Baldwin JN, Ferreira P, Simic M, Vanicek N, Burns J, et al. Normative reference values for strength and flexibility of 1,000 children and adults. Neurology. 2017;88(1):36-43.

6. Merkies IS, Schmitz PI, Samijn JP, Meche FG, Toyka KV, van Doorn PA. Assessing grip strength in healthy individuals and patients with immune-mediated polyneuropathies. Muscle Nerve. 2000;23(9):1393-401.

7. Allen JA, Pasnoor M, Dimachkie MM, Ajroud-Driss S, Brannagan TH, Cook AA, et al. Quantifying Treatment-Related Fluctuations in CIDP: Results of the GRIPPER Study. Neurology. 2021;96(14):e1876-e86.

8. Vanhoutte EK, Latov N, Deng C, Hanna K, Hughes RA, Bril V, et al. Vigorimeter grip strength in CIDP: a responsive tool that rapidly measures the effect of IVIG--the ICE study. Eur J Neurol. 2013;20(5):748-55.
