# Supplementary material for: Assessing hand motor function in chronic immune-mediated neuropathies: a proof-of-concept study using a data glove
Source: J Neuroeng Rehabil. 2024 Dec 20;21:218. doi: 10.1186/s12984-024-01518-3 (PMC11662497; doi:10.1186/s12984-024-01518-3)
Supplement: Supplementary file 4 — Additional file 4. Means of the data glove movement patterns and the other outcome measures. Listed for the four relevant time points, differentiated for the subgroups of patients with and without clinically relevant hand motor impairment. [file 12984_2024_1518_MOESM4_ESM.docx]

**Additional file 4 – Means of the data glove movement patterns and the other outcome measures**

**Additional file 4 – Table 1: Results of the data glove movement patterns for patients with hand motor impairment**

|  | **time point** | | | |
| --- | --- | --- | --- | --- |
| **glove movement pattern** | **T_1_** | **T_2_** | **T_3_** | **T_4_** |
| finger spread (°) | 33.2 (14,3) (n=22) | 32.7 [15.8]  (n=23) | 35.2 (14.3)  (n=22) | 32,7 (16.3)  (n=23) |
| thumb opposition (°) | 85.4 (31.6) (n=25) | 91.6 [36.1]  (n=25) | 92.5 (42.8)  (n=25) | 93.6 (40.8)  (n=24) |
| fist opening (°) | 127.1 [30.7]  (n=25) | 129.2 [36.2]  (n=25) | 139.7 [16.7]  (n=25) | 128.5 (35.9)  (n=24) |

Metric and normally distributed data is listed as mean (SD (standard deviation)), metric non-normally distributed data are given as median [IQR]. All results are shown for the dominant respectively the only affected hand.

**Additional file 4 – Table 2: Results of the data glove movement patterns for patients without hand motor impairment**

|  | **time point** | | | |
| --- | --- | --- | --- | --- |
| **glove movement pattern** | **T_1_** | **T_2_** | **T_3_** | **T_4_** |
| finger spread (°) | 43.1 (7.5) (n=22) | 47.0 [6.7]  (n=23) | 47.2 (7.4)  (n=22) | 46.0 (6.7)  (n=23) |
| thumb opposition (°) | 115.8 (19.3) (n=25) | 117.3 [15.6]  (n=25) | 125.6 (16.4)  (n=25) | 117.8 (14.2)  (n=24) |
| fist opening (°) | 148.6 [10.2]  (n=25) | 154.5 [12.4]  (n=25) | 150.9 [16.8]  (n=25) | 153.3 (19.8)  (n=24) |

Metric and normally distributed data is listed as mean (SD (standard deviation)), metric non-normally distributed data are given as median [IQR]. All results are shown for the dominant respectively the only affected hand.

**Additional file 4 – Table 3: Results of the clinical parameters for patients with hand motor impairment**

|  | **time point** | | | |
| --- | --- | --- | --- | --- |
| **parameter** | **T_1 (_**_n=25)_ | **T_2 (_**_n=25)_ | **T_3 (_**_n=25)_ | **T_4 (_**_n=24)_ |
| Vigorimeter (kPa) | 43.8 (27.7) | 44.4 (27.1) | 43.7 (27.6) | 42.9 (28.1) |
| INCAT (arm sub-score) | 2.5 [1.0] | 2.5 [1.0] | 2.5 [1.0] | 3 [1.0] |
| MRC (arm sub-score) | 28.0 [3.0] | 28.0 [2.5] | 28.0 [2.5] | 28.0 [2.5] |
| R-ODS (logits) | 1.5 (2.5) | 1.5 (2.5) | 1.6 (2.2) | 1.2 (2.2) |
| FSS | 3.8 [3.5] | 3.7 [2.8] | 3.6 [3.7] | 3.0 [3.8] |
| BDI | 7.0 [19.0] | 7.0 [17.8] | 16.8 [17.8] | 17.0 [19.0] |

Metric and normally distributed data is listed as mean (SD), ordinal or metric non-normally distributed data are given as median [IQR]. Results of the Vigorimeter are shown for the dominant respectively the only affected hand. (INCAT = Inflammatory Neuropathy Cause and Treatment, MRC = Medical Research Council, R-ODS = Rasch-built Overall Disability Scale, FSS = Fatigue Severity Scale, BDI = Beck´s Depression Inventory)

**Additional file 4 – Table 4: Results of the clinical parameters for patients without hand motor impairment**

|  | **time point** | | | |
| --- | --- | --- | --- | --- |
| **parameter** | **T_1 (_**_n=25)_ | **T_2 (_**_n=25)_ | **T_3 (_**_n=25)_ | **T_4 (_**_n=24)_ |
| Vigorimeter (kPa) | 89.1 (20.4) | 89.7 (19.9) | 88.2 (19.1) | 88.2 (21.5) |
| INCAT (arm sub-score) | 1.0 [1.3] | 1 [1.0] | 1.0 [1.3] | 1.0 [1.3] |
| MRC (arm sub-score) | 30.0 [0.0] | 30.0 [0.0] | 30.0 [0.0] | 30.0 [0.0] |
| R-ODS (logits) | 3.5 (2.7) | 3.2 (2.4) | 3.1 (2.6) | 2.7 (2.3) |
| FSS | 2.9 [2.5] | 3.2 [3.4] | 2.3 [2.2] | 3.0 [1.8] |
| BDI | 4.0 [6.0] | 4.0 [6.0] | 4.0 [6.5] | 5.0 [6.5] |

Metric and normally distributed data is listed as mean (SD), ordinal or metric non-normally distributed data are given as median [IQR]. Results of the Vigorimeter are shown for the dominant respectively the only affected hand. (INCAT = Inflammatory Neuropathy Cause and Treatment, MRC = Medical Research Council, R-ODS = Rasch-built Overall Disability Scale, FSS = Fatigue Severity Scale, BDI = Beck´s Depression Inventory)

**Additional file 4 – Table 5: Results of the NCS and HRUS parameters for the two subgroups for T_2_ and T_4_**

|  | **Patients with hand impairment (n=14)** | | **Patients without hand impairment (n=11)** | | |
| --- | --- | --- | --- | --- | --- |
| **Parameter** | **T_2_** | **T_4_** | **T_2_** | **T_4_** |  |
| CV median nerve (forearm) (m/s) | 45.0 (11.1)  (n=13) | 44.9 (10.8)  (n=13) | 47.6 (8.0)  (n=11) | 47.8 (7.9)  (n=11) |  |
| CV ulnar nerve (forearm) (m/s) | 48.4 (9.1)  (n=14) | 50.0 (8.2)  (n=13) | 55.8 (8.5)  (n=11) | 55.5 (8.8) (n=11) |  |
| CV superficial radial nerve (forearm) (m/s) | 52.9 [10.9]  (n=14) | 54.1 [10.6]  (n=13) | 53.2 [6.1]  (n=11) | 52.9 [6.0]  (n=11) |  |
| CSA median nerve (upper arm) (mm^2^) | 12 [3.8]  (n=14) | 12 [5.0]  (n=13) | 11 [4.5]  (n=11) | 10 [2.5]  (n=11) |  |
| CSA ulnar nerve (upper arm) (mm^2^) | 9 [5.5]  (n=14) | 8 [5.0]  (n=13) | 9 [4.0]  (n=11) | 10 [2.5]  (n=11) |  |
| CSA radial nerve (radial sulcus) (mm^2^) | 6.5 [1.8]  (n=14) | 6 [2.0]  (n=13) | 6 [4.5]  (n=11) | 6 [4.0]  (n=11) |  |
|  |  |  |  |  |  |

Metric and normally distributed data is listed as mean (SD), ordinal or metric non-normally distributed data are given as median [IQR].
All results are shown for the dominant respectively the only affected hand. (NCV = nerve conduction velocity, CSA = cross-sectional area)
