# Supplementary material for: Assessing hand motor function in chronic immune-mediated neuropathies: a proof-of-concept study using a data glove
Source: J Neuroeng Rehabil. 2024 Dec 20;21:218. doi: 10.1186/s12984-024-01518-3 (PMC11662497; doi:10.1186/s12984-024-01518-3)
Supplement: Supplementary file 5 — Additional file 5. Correlations between the data glove movement patterns and NCV/CSA. Description of the statistics and results of the correlations between the glove movement patterns and nerve conduction velocity at the forearm, respectively, the cross-sectional area at the upper arm of the corresponding nerves. [file 12984_2024_1518_MOESM5_ESM.docx]

**Additional file 5 – Correlations between the data glove movement patterns and the NCV/CSA of the corresponding nerve**

**Statistics:**

Correlation analyses assessed the relationship between the movement patterns of the glove and the NCV at the forearm, respectively the CSA at the upper arm of the upper limb nerve, which mainly mediates the specific movement. The analyses were done for the patients with motor hand impairment at T_2_. For normally distributed metric data, correlations were quantified using Pearson’s correlation coefficient. For ordinal or non-normally distributed metric data, the Kendall-Tau correlation coefficient was applied. Due to the exploratory nature of our trial and the limited number of cases, Bonferroni correction for multiple testing was not applied. The strength of the correlation was interpreted according to Cohen [1].

**Results:**No significant correlations could be revealed (Tab. 1). Additionally, the directions of the correlations were not consistent and sometimes diverged from the clinically expected direction (Tab. 1), as the correlations between NCV and the glove movement patterns should have been consistently negative, and the ones between CSA and the glove movement patterns should have been consistently negative. Finally, mainly weak and one moderate effect size was shown (Tab. 1). However, the strength and direction of the correlations are not truly valid to interpret given the non-significant p-values.
Altogether, there seems to be no relevant clear linear relationship between the CSA at the upper arm, the NCV at the forearm, and the various movement patterns of the data glove.

**Additional file 5 – Table 1: Results of the correlation analyses between the data glove movement pattern and NCV/CSA**

| **Movement pattern of the glove** | **NCV site** | **r** | **p-value** |
| --- | --- | --- | --- |
| finger spread | ulnar nerve forearm | 0.38 | 0.219 |
| thumb opposition | median nerve forearm | -0.24 | 0.423 |
| fist opening | radial superficial nerve forearm | 0.19 | 0.352 |
| **Movement pattern of the glove** | **CSA site** | **r** | **p-value** |
| finger spread | ulnar nerve upper arm | 0.14 | 0.536 |
| thumb opposition | median nerve upper arm | 0.10 | 0.617 |
| fist opening | radial nerve radial sulcus | -0.19 | 0.366 |

* was defined as p < 0.05, n=12 for the correlations of the finger spread movement pattern due to insufficient range of motion, n=14 for the other correlations, except for the correlation: thumb opposition/NCV median nerve forearm: n=13. (NCV = nerve conduction velocity, CSA = cross-sectional area)

**References:**

1. Cohen J. A power primer. Psychol Bull. 1992;112(1):155-9.

2. Kerasnoudis A, Pitarokoili K, Behrendt V, Gold R, Yoon MS. Correlation of nerve ultrasound, electrophysiological and clinical findings in chronic inflammatory demyelinating polyneuropathy. J Neuroimaging. 2015;25(2):207-16.

3. Kerasnoudis A, Pitarokoili K, Behrendt V, Gold R, Yoon MS. Multifocal motor neuropathy: correlation of nerve ultrasound, electrophysiological, and clinical findings. J Peripher Nerv Syst. 2014;19(2):165-74.
