# Supplementary material for: Assessing hand motor function in chronic immune-mediated neuropathies: a proof-of-concept study using a data glove
Source: J Neuroeng Rehabil. 2024 Dec 20;21:218. doi: 10.1186/s12984-024-01518-3 (PMC11662497; doi:10.1186/s12984-024-01518-3)
Supplement: Supplementary file 6 — Additional file 6. Results of the longitudinal data analyses using LMM. Results of the ANOVA for the different clinical outcome measures and the glove movement patterns. P-values of the analyses using the LMM with an interaction term—comparing the slopes of the Vigorimeter and the three different glove movement patterns throughout the study. [file 12984_2024_1518_MOESM6_ESM.docx]

**Additional file 6 – Results of the longitudinal data analyses using LMM**

**
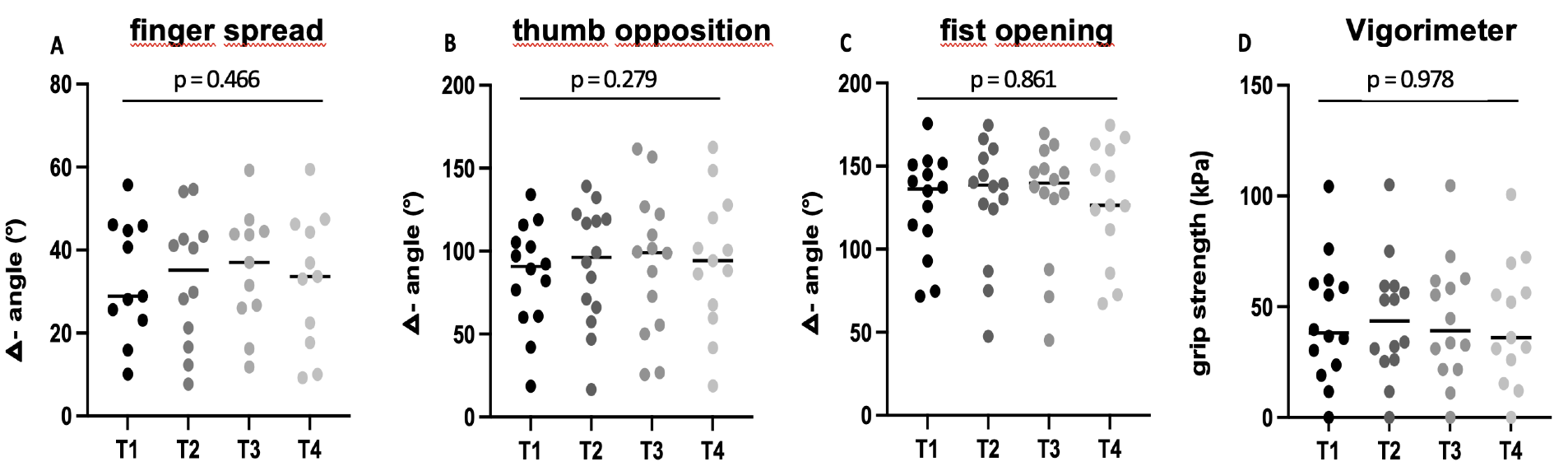
**

**Additional file 6 – Figure 1: Results of repeated measures analyses (Type III ANOVA in R).** The graphics (A-D) show the results of the repeated measures ANOVAs which searched for significant differences throughout the whole study course for each movement pattern of the dataglove and for the Vigorimeter as the primary reference tool. Mean (horizontal line) as well as individual values are shown for each time point. P-values are given as the overall result of the ANOVA. Due to missing data of one patient at T_4_, the number of patients was reduced by one at this time point for every parameter. Moreover, for the finger spread movement pattern, one patient only contributed to the data at T_2_ and T_4_ and two further patients did not contribute to any of the time points due to insufficient range of motion.

**Additional file 6 - Table 1: p-values of repeated measures ANOVA Type III for the glove movement patterns and the Vigorimeter**

| **Parameter** | **p-value** | **F Value** | **(DFn/DFd)** |
| --- | --- | --- | --- |
| finger spread | 0.466 | 0.873 | 3 / 30.000 |
| thumb opposition | 0.279 | 1.331 | 3 / 38.033 |
| fist opening  Vigorimeter | 0.861  0.978 | 0.250  0.068 | 3 / 38.008  3 / 38.044 |

(DFn = degrees of freedom numerator, DFd = degrees of freedom denominator)

**Additional file 6 - Table 2: p-values of repeated measures ANOVA Type III for other clinical outcome parameters**

| **Parameter** | **p-value** | **F Value** | **(DFn/DFd)** |
| --- | --- | --- | --- |
| R-ODS (logits) | 0.957 | 0.1039 | 3 / 37.998 |
| INCAT (arm sub-score) | 0.194 | 1.6522 | 3 / 38.022 |
| MRC (arm sub-score) | 0.335 | 1.168 | 3 / 38.012 |

(DFn = degrees of freedom numerator, DFd = degrees of freedom denominator, R-ODS = Rasch-built Overall

Disability Scale, INCAT = Inflammatory Neuropathy Cause and Treatment, MRC = Medical Research Council)

**Additional file 6 - Table 3: p-values of the LMM - interaction term (time:tool)**

| **Interaction term** | **p-value** |
| --- | --- |
| time: finger spread/Vigorimeter | 0.712 |
| time: thumb opposition/Vigorimeter | 0.294 |
| time: fist opening/Vigorimeter | 0.791 |

The interpretation of the p-values of the LMM-interaction terms had to be done with caution because only little changes occurred for both tools. Moreover, we were only able to demonstrate that there were no significant differences between the trends of the glove movement patterns and the Vigorimeter. Further analyses have to be done, most likely in treatment naive patients, to determine the potential of the data glove to differentiate between clinical stability and relevant changes for confirming the findings of this study.
